# A systematic review of working conditions and occupational health among immigrants in Europe and Canada

**DOI:** 10.1186/s12889-018-5703-3

**Published:** 2018-06-20

**Authors:** T. Sterud, T. Tynes, I. Sivesind Mehlum, K. B. Veiersted, B. Bergbom, A. Airila, B. Johansson, M. Brendler-Lindqvist, K. Hviid, M. -A. Flyvholm

**Affiliations:** 1 National Institute of Occupational Health, Oslo, Norway; 2 Finnish Institute of Occupational Health, Helsinki, Finland; 3 Occupational and Environmental Medicine, Uppsala University Hospital, Uppsala, Sweden; 4 National Research Centre for the Working Environment, Copenhagen, Denmark

**Keywords:** Emigrants and immigrants, Labour migrant, Migrant worker, Occupations, Occupational injury, Occupational safety and health, Review, Systematic review, Work

## Abstract

**Background:**

A systematic attempt to summarize the literature that examines working conditions and occupational health among immigrant in Europe and Canada.

**Methods:**

We established inclusion criteria, searched systematically for articles included in the Medline, Embase and Social Sciences Citation Index databases in the period 2000–2016 and checked the reference lists of all included papers.

**Results:**

Eighty-two studies were included in this review; 90% were cross-sectional and 80% were based on self-report. Work injuries were consistently found to be more prevalent among immigrants in studies from different countries and in studies with different designs. The prevalence of perceived discrimination or bullying was found to be consistently higher among immigrant workers than among natives. In general, however, we found that the evidence that immigrant workers are more likely to be exposed to physical or chemical hazards and poor psychosocial working conditions is very limited. A few Scandinavian studies support the idea that occupational factors may partly contribute to the higher risk of sick leave or disability pension observed among immigrants. However, the evidence for working conditions as a potential mediator of the associations between immigrant status and poor general health and mental distress was very limited.

**Conclusion:**

Some indicators suggest that immigrant workers in Europe and Canada experience poorer working conditions and occupational health than do native workers. However, the ability to draw conclusions is limited by the large gaps in the available data, heterogeneity of immigrant working populations, and the lack of prospectively designed cohort studies.

**Electronic supplementary material:**

The online version of this article (10.1186/s12889-018-5703-3) contains supplementary material, which is available to authorized users.

## Background

According to the International Labour Organization’s estimates, there are 150 million immigrant workers throughout the world, almost half of whom are concentrated in two broad subregions, Northern America and Europe. In Europe, the proportion of foreign-born residents increased by more than 50% in the first decade of 2000 because of mobility and migration, and this group now represents about 10% of the European population [1]. Immigrant workers are commonly defined as all economically active immigrants because most of the data sources cannot define the reasons for migration and are likely to record only nationality or country of birth. Most immigrant workers throughout the world are engaged in the services sector and in industries such as manufacturing, construction, transportation and agriculture [2]. New European Union (EU) and national state policies to liberalize regulations have been introduced during the last decade to open up labour markets in Europe, to stimulate new supply- and demand-driven forms of labour migration, and to meet labour market demands and demographic outlook. Most of the immigrant workers from inside and outside of Europe work in low-skilled jobs [1]. Although both immigrant status and unskilled labour are thought to constitute particular risks of unsafe and unhealthy working environment, relatively little is known about working conditions and work-related health of migrants in host countries [3].

Paid work is important for quality of life because it provides a source of income and identity. The workplace offers opportunities for personal development and socializing [4]. However, not all jobs provide equal opportunities, and some are characterized by occupational hazards such as heavy physical work, risk of injury or exposure to toxic substances or poor psychosocial working conditions (e.g., excessive mental work load, low job autonomy or negative social interactions). It is well documented that such exposures can negatively affect workers’ health [5]. In destination countries, immigrant workers are reported to be over-represented in less desirable, low-skilled jobs and are thought to be more exposed to adverse working conditions than natives [6]. Greater difficulties in entering the labour market and in validating prior educational and technical training once in the host country, poor language skills, and a lack of workers in some unskilled occupations may contribute to the higher rate of immigrant employment in the most hazardous jobs. Hence, there are reasons to assume that work-related health among the immigrant population differs from that of the native population in various countries. Other factors such as the reason for migration, geographical origin, age at migration and residence time in the new country also likely contribute to differences in health status between immigrant groups and the native population [7]; however, these topics were considered to be beyond the scope of the systematic search in present study.

More than 10 years have passed since Ahonen and co-workers published the most recent review of research on occupational health among immigrant groups [8]. Their search strategy captured both original and overview articles relating to the topics of immigration, work and health in the PubMed database for the period 1990–2005. Nearly 90% of the included studies were conducted in the United States, Australia and Canada, while only a few were conducted in Europe. The most studied outcome noted in their review was occupational injuries, whereas studies of exposure and occupational health problems involved mainly specific populations (e.g., farm workers and textile workers). The authors reported that the studies included were highly heterogeneous and difficult to classify. Nevertheless, they concluded that all indicators together drew a worrying image of immigrant workers’ health.

Our objective here was to perform a systematic review of the research on both working conditions and occupational health among immigrant workers in Europe. We included studies from Canada because its immigration regime is similar to that of some European countries, especially the Scandinavian ones. We aimed to compare the relationship between working conditions and occupational health in immigrant and native workers. Our main research questions were as follows:

Research question 1: A) Do differences in working environment and conditions exist? B) Does the relationship between work-related exposure and health differ between these groups?

Research question 2: A) Do immigrant workers have more occupational health problems than native workers? B) Do differences pertaining to working conditions mediate differences in occupational health problems?

## Methods

In this review, we defined “immigrant worker” in a general sense as a person who is foreign-born and economically active in the host country. We chose a wide definition to allow us to examine different aspects of work and health for diverse groups of immigrants or minorities in multiple contexts.

### Search strategy

We searched systematically for the period 2000–2016 in the *Medline, Embase* and *Social Sciences Citation Index* databases during January 2017. We limited the search to article titles and abstracts. We prepared one list of search terms related to immigration, a second related to occupational health or occupational exposure based on the search string suggested by Mattioli and co-workers [9], and a third related to the country of immigration (see Additional file 1). Other relevant sources were identified through the reference lists of all included studies and other relevant studies identified by the authors.

### Inclusion/exclusion criteria and assessment

Two of the authors screened the abstracts and excluded those that did not mention immigrant populations and occupational exposure or occupational health as central issues. All potentially relevant papers were read in full by one of the authors. If exclusion was suggested, it was confirmed by the first author. For inclusion, studies had to meet all the following criteria:

1. The study included and reported data for employed immigrants.
2. The study either addressed a quantitative measure of occupational exposure or the health status of a working population or analysed the relationship between health and working conditions
3. The study was an original study published in a peer-reviewed journal, its abstract was reported in at least one of the databases.
4. The study was published in English or a Nordic language (Danish, Finnish, Norwegian or Swedish).

The included articles were assessed by one of the authors and then the main author using a set of predefined parameters that included the study design, characteristics of the participants, definitions and measurement of working conditions and health, statistical analysis, covariates, results and limitations. This information is summarized in a table (see Additional file 2).

## Results

**Fig. 1:**
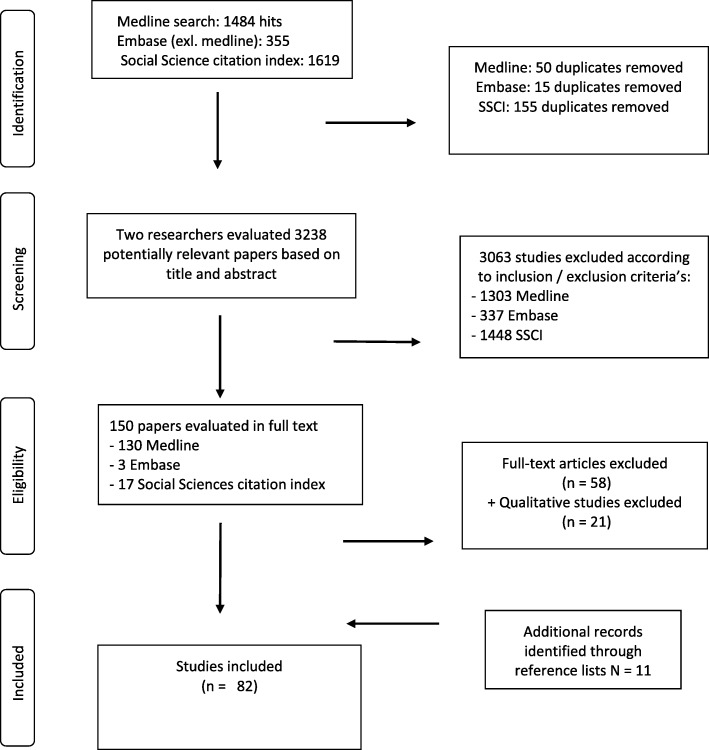
Flow chart

The search resulted in 3213 hits in the three databases after we had removed all duplicates. We excluded most of the studies (*n* = 3063) in the initial screening of titles and abstracts. In total, 151 articles were read in full, 92 of which fulfilled the initial inclusion criteria [10–80]. In addition, 11 studies [81–91] identified in the reference lists were included. The excluded studies that were read in full did not report data on working conditions or health-related outcomes in a defined working population (*n* = 53); three were duplicates, and two were historical studies of asbestos and mesothelioma [92, 93]. Twenty-one studies [94–114] did not report relevant quantitative measures of exposure or health. Thus, 82 studies were included in this review (see the flow chart in Fig. 1).

Most studies were cross-sectional (*n* = 77), except for five with a longitudinal design [26, 45, 62, 70, 81]. Most studies were questionnaire-based surveys (*n* = 66), except for some register-based studies of sick leave or disability pension [22, 28, 37, 42, 45, 73, 81, 82] or work injury [16, 20, 26, 29, 40, 50, 59].

The studies were from Canada (*n* = 13), Czech Republic (*n* = 2), Denmark (*n* = 9), Finland (*n* = 5), Germany (*n* = 2), Greece (*n* = 1), Ireland (*n* = 5), Italy (*n* = 2), the Netherlands (*n* = 2), Norway (*n* = 7), Spain (*n* = 20), Sweden (*n* = 7), Switzerland (*n* = 2), the United Kingdom (UK) (*n* = 4), and Europe (*n* = 1).

### Working conditions and their association with health (*n* = 43 studies)

Of the 43 studies addressing working conditions, 32 addressed research question 1A pertaining to differences in specific work-related exposures and 17 examined research question 1B on whether the relationships between specific exposures and health effects differ between immigrants and natives. These results are grouped into the following categories: mechanical, physical or chemical exposures, psychosocial stressors, bullying or discrimination and different employment arrangements, summarized in separate tables (Tables 1, 2, 3 and 4).

**Table 1:** Mechanical, physical, chemical exposure among immigrants compared with natives

Author (ref number)	Sample, method, country, study period	Observed mean differences or risk estimates, immigrants compared with natives:
Diaz-Serrano et al. [33]	General working pop., survey, Catalonia, 2006	Noise: mean = 1.8 vs.1.7^a^, dust: mean = 1.9 vs. 1.6^a^, heavy loads: mean = 1.8 vs. 1.6^a^
Dunlavy et al. [35]	General working pop, survey, Sweden, 2010–11.	Physical demanding work: ERR^#^ = 1.3 (Latin-American) ^a^, ERR^#^ = 1.4 (other Non-Western)^a^, awkward working postures^NS^
Premji & Lewchuk [60]	General working pop., survey, Canada, 2005–6	Heavy physical workload: ERR^#^ = 1.7 ^a^, toxic substances: ERR^#^ = 0.6 (m) ^a^
Ronda et al. [64]	General working pop., survey, 31 European countries EU, 2004–5	Vibrations: ERR^#^ = 1.4(m) ^a^ /1.4(w) ^a^, noise: ERR^#^ = 1.3(m) ^a^, high temperature: ERR^#^ = 1.3(m) ^a^, heavy loads: ERR^#^ = 1.2(m) ^a^/1.8 (w), painful positions: ERR^#^ = 1.21(m)^a^, standing: ERR^#^ = 1.2^a^, fume/dust: ERR^#^ = 0.55 (w) ^a^
Ronda et al. [63]	General working pop., survey, Spain, 2004–5	Lifting weights^NS^, forced positions^NS^, standing: ERR^#^ = 1.2 (m) ^a^/ 1.3 (w) ^a^, chemical exposure^NS^, temperature: ERR^#^ = 1.8(m) ^a^/ 2.1(w) ^a^, noise^NS^
Smith et al. [70]	Cohort of immigrants, survey, Canada, 2000–01,	Higher physical demands compared to before arrival in Canada: Poor English: OR = 1.7 ^a^, Refugee applicants: OR = 2.9 ^a^.Data on natives = n/a

*OR* odds ratio, *RR* relative risk, ^#^ *ERR* estimated relative risk based on reported prevalence numbers

^a^statistically significant. ^*NS*^ not statistically significant, *m* men, *w* women, *n/a* not available

**Table 2:** Psychosocial work factors among immigrants compared with natives

Author (ref number)	Sample, method, country, study period	Observed mean differences or risk estimates, immigrants compared with natives:
Aalto A-M et al. [10]	Physicians, survey, Finland, 2010	Time pressure: mean = 3.1 vs 3.1^NS^, job control: mean = 4.2 vs. 4.1^NS^, team climate: mean = 3.96 vs. 3.89^NS^, organizational justice: mean = 4.0 vs 3.9^a^
Cross and Turner [30]	A sample of immigrant workers, survey, Ireland, 2006–08	Non-EU immigrants reported more distributive and interactional unfairness at work than EU immigrants. No data for natives.
Dunlavy et al. [35]	General working pop., survey, Sweden, 2010–11.	High demands: PR 46–53% vs 51%, ow decision latitude: PR 43–60% vs 45%, low social support: PR 22–32% vs 25%. No statistical test provided.
Font et al. [39]	General working pop., survey, Spain, 2004/5.	High demands: RR 1.33 ^a^, low influence: RR 2.58 ^a^, low support: RR 1.79 ^a^ (manual workers only)
Hoppe [44]	Employees in a mail service company, survey, Germany, n/a	Time pressure: mean = 3.1 vs 2.9 ^NS^, job control: mean = 2.8 vs 3.0 ^NS^, supervisor support: mean = 2.8 vs 3.0^NS^, conflicts with colleagues: mean = 1.6 vs 1.3 ^a^
Jönson and Giertz [46]	Care workers, survey, Sweden, 2005	High workload: OR 3.3 ^a^, low influence on working conditions: OR 1.35^NS,^low support: OR 0.90^NS^, not appreciated by colleagues OR 2.2 ^a^
Olesen et al. [53]	A sample of cleaners, survey, Denmark, 2007–8	Quantitative demand: OR 0.67^NS^, influence (control): OR 0.64^NS^, social support from colleagues: OR 0.84^NS^, social support from supervisor OR 1.21^NS,^ quality of leadership: OR 1.81 ^a^
Ortega et al. [55]	Elderly care workers in 36 Municipalities, survey, Denmark, 2005	Workload: mean = 48.1vs 47.1^NS^, influence: mean = 56.6 vs 44.9 ^a^, development: mean = 67.8 vs 72.2 ^a^, leadership quality: mean = 59.8 vs 56.2^NS^
Ronda et al. [64]	General working pop., survey, Europe, 2005	Work pace: RR = 1.23 ^a^, shift work: RR = 1.66 (non-maual workers) ^a^, long working hours (> 10 h day): RR = 1.09^NS^
Ronda [63]	General working pop., survey, Spain, 2004–5	Work pace: ERR^#^ = 1.01^NS^ (m) and ERR^#^ = 1.11^NS^ (w), long working hours: ERR^#^ = 1.35 ^a^ (m) and ERR = 1.46 ^a^ (w)
Sundquist et al. [77]	General working pop., survey, Sweden, 1994–97	Low decision latitude: PR 63% (refugee manual workers) vs. 45% (natives). Small differences in job demands and social support. No statistical test.
Tora et al. [80]	General working pop., survey, Spain, 2007	Job demands: mean = 44 vs 52, low job control: mean = 60 vs 49, low social support: mean = 52 vs 47. No statistical test.

*PR* prevalence (%), *OR* odds ratio, *RR* relative risk, ^#^*ERR* estimated relative risk based on reported prevalence numbers

^a^statistically significant. ^*NS*^ not statistically significant, *m* men, *w* women, *n/a* not available

**Table 3:** Bullying (B) or discrimination (D)

Author (ref number)	Sample, method, country, study period	Observed mean differences or risk estimates, immigrants compared with natives:
Akhavan et al. [15]	Women working in a municipality, survey, Sweden, 2003	(D): OR 2.90, 90%CI 2.23–3.76 (PR = 14% vs 6%)
Bergbom et al. [18]	Employees at a bus company, survey, Finland, n/a	(B): OR 3.4, 95%CI 1.8–6.6 (PR =21.8% vs 7.6%)
Bhui, et al. [19]	General working pop., survey, UK, 1998–99	(D): ERR^#^ = 1.7^a^, (PR = 11% vs 6.6%)^a^
Diaz-Serrano [33]	General working pop. of immigrants, survey, Spain, 2006	(D): PR = 28% (Africans), 14.4% (Latin-Americans), and 4.9% (EU15)
Dzurova and Drbohlav [36]	General working pop., survey, Czech Republic, 2013	(D): ERR^#^= 7.3 (29% versus 4%)^a^ (m) and ERR^#^ = 5.4 (PR = 38% vs. 7%)^a^ (w).
Gil-Gonzalez et al. [41]	General working pop., survey, Spain, 2006–7	(D): OR 48.1, 95%CI 28.2–82.2 (PR = 5.7% vs 0.1%)^a^ (m) and OR 43.5 95%CI 25.5–74.3(PR = 0.1% vs. 5.0%)^a^ (w)
Hogh et al. [43]	Health care students/workers, survey, Denmark, 2004	(B): OR 1.85, 95%CI 1.20–2.87 (PR = 15.2% vs 8.5%)
Jönson and Giertz [46]	Care workers, survey, Sweden, 2005	(D): OR 1.66^a^
Krings et al. [48]	General working pop. Survey, Switzerland, 2012	(D): OR 13^a^ (German/French) and OR 7.3^a^ (another nationality)
Miller & Travers [51]	Teachers, survey, UK, n/a	(D): mean = 107.7 vs mean = 101.5^a^
Shields and Price (86	Nurses, survey, UK, 1994.	(D): PR = 6.5% (staff) and 9.7% (colleagues). No data for natives.
Wadsworth et al. [91]	General working pop., survey, UK, 1998–99	(D): ERR^#^ = 2 (African–Caribbean) and ERR^#^ = 1.2 (Bangladesh). No statistical test.

*PR* prevalence (%), *OR* Odds ratio, ^#^*ERR* estimated relative risk based on reported prevalence numbers

^a^statistically significant. ^*NS*^ not statistically significant, *m* men, *w* women, *n/a* not available

**Table 4:** Employment conditions among immigrants compared with natives

Author (ref number)	Sample, method, country, study period	Observed mean differences or risk estimates, immigrants compared with natives:
Akhavan et al. [15]	Women working in a municipality, survey, Sweden, 2003	Temporary contract: PR 20% vs 8% (m) (no statistical test provided)
Borrell et al. [21]	A sample of immigrant workers, survey, Ireland, 2006–08	Temporary contracts: PR 40% vs 27%^a^ (m) and PR 19% vs 21%^NS^ (w)
Chen et al. [26]	General working pop. of immigrants, survey, Canada, 2001–4.	Over-educated: PR 52% (PR range: 32% western Europe thru 63% Southeast Asia). No data on natives.
Diaz-Serrano [33]	General working pop., survey, Spain, 2004/5.	Permanent contract: Prevalence difference = −33% (Latin American) ^a^ and − 38% (African) ^a^ and − 7% ^a^ (EU15)
Dunlavy et al. [34]	General working pop., Sweden 2010	Over-educated (objectively): PR 21% (non-western) and PR 15% (Western Europe) vs. PR 14% among native-born workers. No statistical test.
Premji and Lewchuk [60]	General working pop. Survey, Canada, 2005–6	Temporary contract: PR 37% vs 42% ^NS^
Ronda et al. [64]	General working pop., survey, 31 European countries EU, 2004–5	No work contract: PR 10% vs 7% ^a^ (m) and PR 17% vs 8% (w)^a^
Smith PM and Mustard [72]	General working pop., survey, Canada, 2001	Temporary contract: OR 1.84, 95%CI 1.04–3.26
Solé et al. [73]	General working pop., survey, Spain, 2006	Temporary contracts: 48% vs. 37% ^a^
Sousa et al. [75]	General working pop., survey, Spain, 2008–9.	Work contract: PR 41% temporary, 9% no contract, 24% undocumented vs. PR 41% and 12%, n/a, respectively.
Vives et al. [90]	General working pop., survey, Spain, 2004/5	Employment precariousness: PR 18.3% vs 5.6% ^a^

*OR* Odds ratio, *PR* prevalence (%)

^a^statistically significant. ^*NS*^ not statistically significant, *m* men, *w* women, *n/a* not available

#### Mechanical, physical or chemical exposure and health (*n* = 6 studies; Table 1)

A study from 31 European countries, compared immigrant workers with natives and found that immigrant manual workers reported higher levels of exposure to physical factors (vibrations, noise and heat) and mechanical factors (painful positions, heavy loads and standing or walking). Exposure to dust or fumes was more prevalent among female immigrant workers only [64].

Three national surveys that compared immigrant workers to natives reported greater exposure to heavy physical demands [33, 35, 60], and two surveys reported small and non-significant differences for lifting weights and forced work position [63] and working postures [35]. Surveys from Spain reported greater exposure to dust among immigrant workers [33], but no significant differences for chemical exposure [63]. A survey from Canada reported lower exposure to toxic substances for immigrants [60]. A second survey from Canada reported that, both 2 and 4 years after arrival, immigrants with poorer English language skills or lower educational level or those who had immigrated to Canada as a refugee were more likely to be employed in occupations with greater physical demands compared with their previous jobs before arriving in Canada [70].

#### General psychosocial working conditions and health (*n* = 18 studies; Table 2)

Three studies reported greater job demands among immigrants [39, 46, 64], while one reported lower job demands [80], and six reported small and no significant differences between natives and immigrants [10, 35, 44, 53, 55, 63]. Four studies of the general population reported lower levels of job control in immigrant workers [35, 39, 77, 80], whereas three studies of workers within the same occupation found no significant differences between immigrants and natives [10, 44, 53], and one study reported a significant higher level of job control among immigrants [55]. Two studies of the general population [39, 80] found lower levels of social support among immigrant workers, whereas a third study of the general population found no differences [35]. Three studies that compared immigrants and natives within the same occupation found no differences in the level of social support from colleagues [44, 53] or perceived leadership quality [55].

Pertaining to research question 1B, similar associations between psychosocial factors and measures of psychological distress were reported for immigrants and natives in three studies of the general working population in Spain [38], employees in a transportation company in Finland [17] and the general working population of Swedish women [87]. By contrast, stressors were more strongly associated with measures of psychological distress among natives than among immigrants in a German study of workers in a mail service company [44], two Danish studies of cleaners [54] and elderly care workers [55] and a Finnish study of physicians [49].

#### Bullying or discrimination in the workplace and health (*n* = 12 studies; Table 3)

Non-Western immigrant health care workers [43], and immigrant employees in a transportation company [18], were more likely to report bullying than natives. Higher levels of perceived discrimination among immigrant workers compared with natives have been observed in studies of the general working population in Spain [33, 41], the Czech Republic [36], Switzerland [48], and the UK [19, 91], and in UK studies of ethnic minority nurses and teachers [51, 86], and in Swedish studies of immigrant women employed in a municipality [15] and non-Nordic immigrants employed in elderly care [46].

Pertaining to research question 1B, a Spanish survey reported an association between work-related discrimination and poor mental health and self-reported health (SRH) among immigrant workers [13]. A study of the general working population in the UK reported that the risk of mental disorders was highest among people from ethnic minorities who reported having received unfair treatment or racial insults [19].

#### Employment conditions and health (*n* = 10 studies; Table 4)

Studies of the general working population from Sweden [15] and Spain [21, 33, 73], have found that immigrants were more likely to report having a temporary work contract, or to be undocumented and working without a contract [75], whereas studies from Canada have found that recent immigrants were more likely to report temporary employment than were natives [60, 72]. Employment precariousness (i.e., employment instability, low wages, limited rights) was significantly higher among immigrants than among Spanish natives [90]. Over-education, which is defined as a discrepancy between a person’s educational attainment and the educational requirements of his or her occupation, was reported to be more prevalent among workers from outside of Western Europe, compared with natives in the general working population in Sweden [34].

Pertaining to research question 1B, having no work contract or a temporary contract [75] or precarious work situation [89] were all associated to the same extent with poor SRH and mental health in both immigrant and native Spanish workers. Being employed in a temporary job was more strongly related to having disability pension among Spanish natives than among immigrants [73], but was more strongly related to sickness presenteeism among immigrants than among natives [12]. A higher risk of poor mental health was observed among immigrants with illegal or temporary legal status compared with those who had acquired Spanish citizenship [62]. Over-educated foreign-born workers from countries outside Western Europe had double the risk for poor SRH compared with over-educated native-born Swedish workers [34], and 4 years after arrival in Canada, immigrants experiencing any dimension of over-qualification were significantly more likely to report a decline in mental health [26], and had a higher risk of work injuries requiring medical attention compared with non-recent and not over-educated immigrants [61].

### Health problems, sick leave, disability and work injuries (*n* = 45 studies)

Studies addressing whether the prevalence of health problems is higher in immigrant workers than in native workers (research question 2A) have evaluated the following health indicators: SRH and mental distress (*n* = 17), sick leave or disability pension (*n* = 12) and work injuries (*n* = 16). Among the 45 studies, nine examined whether differences pertaining to working conditions mediate the association between immigrant status and health problems [25, 35, 52] or sick leave and disability rates [23, 24, 28, 42, 73, 82] (research question 2B).

#### Self-reported health (SRH) and mental distress (n = 17 studies; Table 5)

A higher risk of poor SRH among immigrants compared with natives, have been reported in general working population studies in Sweden [35], Norway [82] and Spain [21, 25], and studies of cleaners [47] and elderly care workers [23] in Denmark. A study of the general working population from the Czech Republic reported small differences in SRH between natives and immigrants [36]. Two studies compared SRH between groups of immigrant workers [58, 76].

**Table 5:** Self-reported health (SRH) and mental distress

Author (ref number)	Sample, method, country, study period	Observed mean differences or risk estimates, immigrants compared with natives:
SRH		
Borrell et al. [21]	General working pop., survey, Spain. 2001–01	OR 2.16, 95%CI 1.14–4.10^a^ (m) and OR 1.15, 95%CI 0.59–2.23^NS^ (w)
Brekke et. Al [82]	General working pop., survey and register data, Norway, 2000–01	ERR^#^ = 2.67 (PR 32% vs. 12%) (m) and ERR^#^ = 2.58 (PR 43 vs. 16%) (w). No statistical test.
Carneiro. et al. [23]	Elderly care workers, survey, Denmark, 2005.	ERR^#^= 1.69 (PR 6.4% vs. PR 10.8%) ^a^
Cayuela et al. [25]	Immigrants born in low-income countries, survey, Spain, 2011/12.	OR 2.64, 95%CI 1.77–3.93 ^a^ (w) and OR 1.33, 95%CI 0.85–2.08 ^NS^ (m)
Dunlavy and Rostila [35]	General working pop., survey, Sweden 2010–11.	OR 2.39, 95%CI 1.74–3.28 (EE), OR 1.50, 95%CI 1.06–2.12 (LA), OR 1.79, 95%CI 1.34–2.40 (N-W)
Dzurova and Drbohlav [36]	General working pop., survey, Czech Republic 2008 and 2012–13.	ERR^#^ = 1.09 (PR = 28% vs. 26%)^NS^ (w), ERR ^a^ = 0.96 (PR 21% vs. 22%)^NS^ (m)
Jørgensen et al. [47]	Cleaners, survey, Denmark 2007–09	ERR^#^ = 1.21 (PR 46% vs. 38%) ^a^
Pikhart et al. [58]	Immigrant workers, survey, Czech Republic 2003/06	No significant differences between illegal and legal immigrants. No data for natives.
Subedi and Rosenberg [76]	immigrants, survey, Canada, 2001 and 2010	Sign. difference in the SRH of immigrants with < 10 years vs. > 10 years of residency in Canada
Mental Health		
Aalto et al. [10]	Elderly care workers, survey, Finland, 2010	Burnout: OR 1.46, 95%CI 1.16–1.85.
Bhui, et al. [19]	General working pop, survey, UK, 1998–99	Poor mental health: PR 12–17% vs. PR 15%^NS^
Cayuela et al. [25]	Immigrants born in low-income countries, survey, Spain, 2011/12.	Poor mental health: OR 2.02, 95%CI 1.39–2.93 (w) and OR 1.43, 95%CI 0.92–2.24 (m).
DelAmo et al. [32]	General working pop., survey, Spain, 2006/07.	Poor mental health: OR 1.6, 95% CI: 1.1–2.4 (w) and OR 1.1, 95%CI 0.7–1.9 (m)
Dunlavy and Rostila [35]	General working pop. Sweden 2010–11.	Poor mental health: OR 2.03, 95%CI 1.39–2.97 (EE) and OR 1.81 (1.22–2.69) (LA)
Font et al. [38]	General working pop, survey, Spain, 20,004/5.	Poor mental health: RR 1.09, 95%CI 1.02–1.16
Gamperiene et. Al. [84]	Female cleaners, survey, Norway, n/a.	Poor mental health: OR 2.8 ^a^
Hoppe [44]	Employees from a mail service company, survey, Germany, n/a.	Psychological job distress: mean = 1.88 vs 1.89^NS^
Niewenhuijsen et al. [52]	General working pop., survey, Netherlands, 2011–15.	Depression symptoms: ERR^#^ = range 1.2 thru 3.2 ^a^
Ortega et al. [55]	Elderly care workers, survey, Denmark, 2005.	Depression symptoms: mean = 8.3 vs. 6.1 ^a^
Pasca and Wagner [56]	Empoyees in health care, and social services, Canada, n/a.	Somatic distress: mean = 51.8 vs 57.5 ^a^
Sieberer et al. [69]	General working pop., survey, Germany, 2008	Poor mental health: OR 2.10, 95% CI: 1.44–3.04
Sundin et al. [87]	A general working pop. Only women, survey, Sweden, 2003	Burnout: mean = 3.2 vs. 3.0 ^a^
Vives et al. [89]	A general working pop., survey, Spain, 2004/5	Poor mental health: ERR^#^ = 1.54 (PR 33% vs 22%) (w) ^a^ and ERR^#^= 1.13 (PR 33% vs. 29.%) (m)^NS^

*OR* Odds ratio, *RR* relative risk, *PR* prevalence (%), ^#^*ERR* estimated relative risk based on reported prevalence numbers, *EE* Eastern Europe, *LA* Latin America, *range* estimates across several groups

^a^statistically significant. ^*NS*^ not statistically significant, *m* men, *w* women, *n/a* not available

Four surveys of the general working population in Spain reported higher risk of mental health problems among immigrant women [25, 32, 89] or both immigrant men and women [38] compared with natives. Higher levels of mental health problems were also found among immigrants in surveys of the general working population in Sweden [35] and the Netherlands [52], a study of hospital employees in Germany [69] and a study of cleaners in Norway [84]. Three studies have reported higher levels of burnout among groups of immigrant workers compared with natives [10, 55, 87]. However, three other studies observed no significant increase in the risk of mental distress in immigrant workers [19, 44, 56].

Pertaining to research question 2B, differences relating to psychosocial working conditions and physical load were reported to have a small or negligible effect on the risk of poor mental health or SRH among immigrants in a study of the general working population in Sweden [35] and among immigrant women in the general working population in Spain [25]. In a study of the working population in the Netherlands, lack of recovery opportunities at work, but not perceived work stress, accounted in part for higher levels of mental health problems in ethnic minority groups compared with natives [52]. In a Norwegian study of female cleaning personnel, adjustment for psychosocial and organizational working conditions did not reduce the observed difference in mental distress between natives and immigrants [84].

#### Sick leave and disability pension (*n* = 12 studies; Table 6)

Four studies of the general working population in Norway [22, 42, 82, 83] and Sweden [81] showed that non-Western immigrants had more general sickness absence [42, 81–83] and pregnancy-related sick leave [22]. However, compared with Norwegian natives, immigrant men from North America and Oceania had lower sickness absence rates, and second-generation immigrants had similar sickness absence rates [83]. Two studies from Denmark reported that immigrants had similar [24] or lower [23] rates of sick leave than natives within the same occupation. A Spanish follow-up study of native and immigrant patients treated by primary care physicians, observed a lower risk of sick leave among immigrants [74].

**Table 6:** Sick Leave and Disability Pension

Author (ref number)	Sample, method, country, study period	Observed mean differences or risk estimates, immigrants compared with natives:
Sick leave		
Bengtsson et al. [81]	General working pop., Register panel data, Sweden, 1982–91	Sick leave (25 days): RR 2 to 7 ^a^ times higher risk
Brekke et al. [82]	General working pop., survey and register data, Norway, 2000/1	Sick leave days: mean 6.3 days more ^a^ (m), mean 8.3 days more ^a^ (w).
Brekke et al. [22]	Cohort of pregnant women, register data 2008–10	Number of sickness absence > 2 weeks: Marginal mean 2.0, 95%CI 1.23–2.77)
Carneiro et al. [23]	Elderly care workers, survey, Denmark, 2005	Sickness absence (≥21 days): RR 0.66 95%CI 0.43–1.01^NS^
Carneiro et al. [24]	Convenience sample Cleaners, survey, Denmark, 2007/8	6-month period: mean 6.7 vs. 5.0 days sick−leave.^NS^
Dahl et al. [83]	General working pop., Register data, Norway, 1992–2003	≥14 days: Asia: OR 1.5 ^a^, Africa OR 1.7 ^a^, North-America OR 0.6 ^a^
Hansen et al. [42]	General working pop., Register, data Norway, 2003–06	≥16 days: Probability 1.3 to 3.6% higher ^a^, mean 1.4 to 3.2 days longer
Soler-Gonzales et al. [74]	Sample of Patients treated in primary care, Spain, 2005	Any period of sick-leave: Natives vs Immigrants RR 1.7 95%CI 1.43–2.02
Disability pension		
Clausen et al. [28]	General working pop., survey and register data, Norway, 2001–2004	OR 2.27, 95%CI 1.55–3.23
Elders et al. [37]	Dutch comparative registry study, Turkish scaffolders, 1981–2000	RR 2.48, 95%CI 1.94–3.18
Johansson et al. [45]	General working pop, register data, Sweden, 2005	HR 1.9, 95%CI 1.9–2.0 (m), HR 1.7, 95%CI 1.7–1.7 (w)
Solé et al. [73]	4% random sample drawn from a Spanish national register	RR 0.3 (PR 1.6% vs. 4.9%)^a^

*OR* Odds ratio, *HR* hazard ratio, *RR* relative risk, *PR* prevalence (%), ^#^*ERR* estimated relative risk based on reported prevalence numbers

^a^statistically significant.^* NS*^ not statistically significant, *m* men, *w* women, *n/a* not available

Nationwide register-based studies of the Swedish [45] and Norwegian [28] working population showed almost double risk of disability pension among immigrant workers compared with natives, and a study from the Netherlands reported a more than double risk of disability pension among Turkish scaffolders compared with natives in the same occupation [37]. By contrast, a nationwide study from Spain reported that immigrants had a lower probability of receiving disability pension than natives [73].

Pertaining to research question 2B, adjustment for occupation (4-digit code) in two studies of the general working population in Norway reduced the observed higher risk of sickness absence among immigrants compared with natives by 12% (in Eastern European immigrants) to 26% (in African immigrants) [42]. Adjustment also decreased the difference in the average number of days on sick leave between immigrants and natives by about one-third [82]. A study from Norway reported that the observed excess risk of using disability pension was largely explained by work factors and level of income, but not by country of origin [28]. By contrast, a study from Spain reported a lower risk of use of disability pension among immigrants despite the worse working conditions for immigrants [73].

#### Work-related injuries (*n* = 16 studies; Table 7)

A higher risk of fatal accidents in immigrants was reported in one study of insured workers in Spain (RR = 4.4; 95% CI 3.9–5.1 in women and RR = 6.0; 95% CI 3.6–9.6 in men) [14]. A higher risk of non-fatal accidents in immigrants was reported in two register-based population studies in Spain and Denmark, respectively [14, 20]. Three survey studies of general working populations found that, compared to natives, the occurrence of self-reported occupational injuries was significantly higher in male immigrants in Italy [67]; immigrant men in their first 5 years in Canada [71]; and immigrant workers in high-risk occupations in Canada [88]. By contrast, a Finnish survey of bus drivers reported a higher injury rate for Finnish than for immigrant drivers [66]. Two studies from Canada using aggregated injury data at the occupational level reported conflicting results in regard to whether immigrants were overrepresented in high-risk occupations [59, 78].

**Table 7:** Non-fatal work injuries among immigrants compared to natives

Author (ref number)	Sample, method, country, study period	Observed differences, immigrants compared with natives:
Ahonen and Benavides [14]	Recorded injuries. General working pop., Spain, 2003	Non-fatal injuries: RR 3.9, 95%CI 3.9–3.9(m) and RR 5.4, 95%CI 5.4–5.5 (w).
Alexe DM et al. [16]	Farm injuries, database run by four major hospitals, Greece, 1996–2000	PR = 23% of the injuries ended with hospitalization vs 14% among Greek farm worker
Biering, et al. [20]	Recorded injuries. General working pop., Denmark, 2003–2013	OR 0.93, 95%CI 0.87–1.00 (old EU and Western) OR 1.13, 95%CI 1.02–1.24 (new EU countries) OR 1.56, 95%CI 1.48–1.64 (rest of the world).
Connel et al. [29]	Patients with eye injuries at an accident and emergency clinic, Ireland, 2006–8	48% of all injuries observed among immigrants their proportion of the work-force was 9%).
Davidson and Orr [31]	Case study of plastic surgery patients, Ireland, 2006/7	40% of all injuries observed among foreign nationals.
Frickman et al. [40]	Emergency department data, Switzerland, 2001–11	66% of all injuries observed among immigrants (> twice the proportion of foreigners in the pop.).
Gravseth et al. [85]	Patients’ records from an Accident and Emergency department, Norway, 2001	30% of all injuries observed among immigrants (their proportion of the work-force was 12%)
Manstrangel et al. [50]	Patients records from an Accident and Emergency department, Italy, 2004	ERR^#^ = 1.68 (109.1 per 1000 compared with 65 per 1000 among Italians)^a^
Premji et al. [59]	General working pop., aggregated work injuries by occupation, Canada, 2000–2	Ta = 0.08^NS^ (% immigrants) / Ta = 0.16^a^ (% recent immigrants)
Saeed et al. [65]	Patients admitted with ocular trauma in Ireland, 2001 and 2006–7	ERR^#^ = 13.4 (134 per 100.000 vs 10 per 100.000 natives)^a^
Salminen et al. [66]	Self-reported injury among bus drivers, Finland, 2005–6	ERR^#^ = 0.68 (77.5 per 1000 employees 113.6)^NS^
Salvatore et al. [67]	Self-reported work injuries in the general working pop., Italy, 2007	RR 1.82, 95%CI 1.53–2.16 (m) and RR 1.20, 95%CI 0.81–1.79 (w)
Sattler et al. [68]	Hand injuries presenting to the Dep. Of Plastic Surgery, 2000–05, Ireland	The patient numbers from the new EU countries increased from 18 (2.4%) to 41 (4.9%).
Smith and Mustard [71]	Self-reported work injuries, general working pop., Canada, 2003 and 2005	OR 2.08, 95%CI 1.02–4.2.5
Thurston and Verhoef [88]	Self-reported work injuries, convenience sample of immigrants,., Canada, 1994	ERR^#^ = 1.70 (Lost-time injury 6.0% of person years worked vs 3.6%. among natives).
Tiagi [78]	Work injuries by occupation in the General working pop. in Canada, 2011	ERR^#^ = 0.97, 69 vs 71 per 10,000^NS^

*OR Odds ratio, RR relative risk, PR prevalence (%), ERR*^a^ estimated relative risk based on the reported prevalence of incidence number reported in the paper, *Ta* Kendall’s Tau, *PR* prevalence (%)

^a^statistically significant. *NS* not statistically significant, *m* men, *w* women, *n/a* not available

Six studies reported that immigrants are over-represented in register-based studies of patients treated for work injuries [29, 31, 40, 50, 65, 85]. The injury rates in immigrants ranged from 109.1 to 271.8 per 1000 non-EU illegally employed people compared with 65 per 1000 for the general working population in Italy in 2004 [50]. A Swiss study of emergency unit patients reported that 66.4% of the injured workers were foreigners; this rate was twice that for the overall proportion of foreigners in Switzerland [40]. The incidence of hospitalized ocular injuries per 100,000 was 134 in immigrants from the EU accession states versus 10 in those of Irish origin in 2006–2007 [65], and the number of patients with a hand injury originating from the 10 new EU accession states in 2004 was reported to increase markedly from 2000 to 2005. Two studies of patients with construction-related eye injuries [29] and workplace injuries requiring referral to a plastic surgery service [31] reported that 48 and 40% of the injuries, respectively, were in foreign-born workers; these workers represented 9% of the total workforce in Ireland. [16]. A Norwegian study of occupational injuries registered in an emergency ward reported that 30% of those with serious injuries had a non-Scandinavian language as their first language; these workers represented 12% of the workforce [85].

## Discussion

The aim of the present paper was to use a systematic approach to explore the literature and determine whether working conditions and occupational health differ between immigrant and native workers in Europe and Canada.

The most robust result in the present analyses is the higher risk of work injuries in immigrant than in native workers in studies from different countries and with different designs (e.g., occupational injury records, national surveys and patient records) [14, 20, 29, 31, 40, 50, 65, 67, 71, 85, 88]. However, one study that compared immigrants and natives with similar jobs and work tasks (bus drivers) did not find a higher risk among immigrants [66]. Different study designs and the fact that many of the studies were based on patient samples without access to the population at risk make it difficult to compare the risk estimates in all studies. Register-based population studies are considered the gold standard for estimating injury rates in the general population; however, a common limitation in all the included studies was that these studies did not account for illegally employed workers, as well as legally workers, who were not found in the national registries. Nevertheless, our findings are consistent with the results from two previous reviews based primarily on studies from the United States (U.S.) [8, 14]. Preventing work injuries in immigrant workers should take a high priority at both the government and enterprise levels.

Across a large number of survey studies, our analyses consistently show that the prevalence rates of bullying [18, 43] and perceived discrimination [15, 19, 36, 41, 46, 48, 51, 86, 91] were higher in immigrants than in natives. However, the different definitions and measures of bullying and discrimination used in these studies rules out the possibility of comparing prevalence estimates. Immigrants do not generally appear to experience poorer psychosocial working conditions than natives within similar occupational groups, and psychosocial working conditions appear to be equally important for health in both immigrants and natives [17, 38, 44, 49, 54, 55, 87]. Nevertheless, results of studies of the general working population show that immigrants are more likely to be employed in jobs with a lower level of autonomy and opportunities for development [35, 39, 77, 80]. In addition, employment conditions such as temporary work [15, 21, 33, 73], lack of work contracts [33] and over-qualification [34] are prevalent and may be important work factors to take into account, especially in studies of recent immigrants [26, 72]. Further studies are needed to replicate these results in different countries and groups of immigrants.

Only a few studies have addressed the physical and chemical working environment of immigrant workers. We did not identify any studies of the health consequences related to physical and chemical exposures in the workplace. Such health consequences may manifest several years after the exposure and are therefore not straightforward to investigate, which may partly explain the lack of studies in this field. A previous review reported that studies of exposure and health problems tended to focus on specific exposure in specific occupational groups, such as pesticide exposure among agricultural workers [8]. However, these studies were conducted in the U.S. Thus, the present study shows that physical or chemical exposures among immigrant workers have been neglected in the European research literature. One possible explanation is that studies of exposure to physical or chemical factors at work may have focused on the exposure and effect in certain occupational groups, as in the U.S., without reporting other characteristics of the exposed groups, such as immigrant status.

Our study shows that immigrant workers report higher levels of poor SRH [21, 23, 25, 35, 47, 82] and mental distress [10, 25, 32, 35, 52, 55, 69, 84, 87, 89] than do natives, which is consistent with the findings of two previous reviews [115, 116]. Our analysis also showed that most [28, 37, 42, 45, 81–83] but not all studies [23, 24, 73] have reported a higher risk of sick leave and disability pension among immigrants compared with natives. The evidence that occupational factors may partly contribute to the excess risk of sick leave and disability pension observed among immigrants is sparse, although a few Scandinavian studies support this observation [28, 42, 82]. However, differences pertaining to working conditions were reported to have a small or negligible impact on the increased risk of poor mental health or SRH among immigrants compared with natives in studies from Scandinavia [35, 84], Spain [25] and the Netherland [52].

### Methodological shortcomings in the primary articles

Our systematic review indicated a need for more high-quality epidemiological studies investigating the relationship between working conditions and occupational health; that is, there are few prospective cohort studies that take various workplace characteristics, immigrant status and baseline health into account.

Most of the included studies of immigrant workers were cross-sectional and relied on self-report. Although self-reported data are an important source of information about the working environment and health in the population, both cognitive and situational factors may influence the validity of the data. Several of the studies used non-validated instruments to measure work exposure or provided little information about the items or instruments used to measure the variables of interest. Moreover, different factors (e.g., language barriers and differences in semantic meanings, expectations and frames of reference) can influence how immigrants evaluate or assess their work environment and understand and interpret the questions and survey context. In addition, a lack of consistency in the assessment methods and instruments make it difficult to compare risk and prevalence across studies of immigrant workers in different study contexts.

Another important consideration is the representativeness of the samples recruited. Immigrants are a heterogeneous group, and individual immigrants may come from different countries, migrate for different reasons, live in different recipient countries and work permanently or for a limited period. Over-sampling is often required to yield sufficient statistical information, and many studies have included small sample sizes that may not have been drawn randomly. Moreover, the lack of access to some populations, such as immigrant workers on short stays or undocumented migrants, is another obstacle.

Most studies of immigrant workers’ occupational exposures and health evaluated in our review focused on differences between immigrants and the native population in the host country; these provide some insights into differences and similarities in occupational exposure and present health status. However, factors such as the diversity of immigrants in terms of their age, sex, country of origin and destination, socio-economic status, the type of migration influence the possibility to perform simple comparisons of the occupational health status between immigrants and natives [7, 117]. Moreover, the “healthy immigrant effect” hypothesis suggesting that migrants are initially healthier than non-migrant populations due to the selection of healthy migrants at migration, but later deterioration of effect because of exposure to risks in host countries, further complicates this issue [117, 118] . Thus, the lack of prospective studies that have included factors that can affect health at different stages before, during and after migration limits the ability to determine the extent to which factors in the work environment, together with other risk factors, may contribute to the risk of illness and disease.

### Limitations and strengths of the current review

Few studies have evaluated the occupational health risks of immigrant populations. This is the first systematic review to summarize the literature on all aspects of working conditions and occupational health in immigrant workers in Europe and Canada. We searched the literature using a number of databases and hand searched the reference list of all the included studies to minimize the risk of missing important studies. The selection of articles in English or Nordic languages and our strict inclusion criteria of original, quantitative, peer-reviewed studies may have led us to overlook relevant documentation published in reports, books or websites that may shed light on this topic. Importantly, the study population in this review represents a narrow spectrum of socio-economic and cultural environments, which makes it impossible to generalize the results to immigrant workers in all parts of Europe or in other parts of the world.

One limitation of this review is the heterogeneity of the methodology used in the included studies. Large differences were observed between the studies in terms of sample size, recruitment methods and assessment of working conditions and occupational health, and these variations restrict our ability to compare and combine the findings of individual studies. Hence, when accounting for the large number of studies with different study aims, populations and methodological approaches, the results will inevitably be a simplification, summary and selection of information and knowledge. Nevertheless, we believe that some general conclusions can be drawn based on the current knowledge about the working conditions and health of immigrants.

## Conclusion

The overall evidence to show that immigrant workers are more exposed to physical or chemical hazards and poor psychosocial working conditions than natives in Europe and Canada is very limited. Nevertheless, the prevalence of bullying and perceived discrimination is consistently higher among immigrant than among native workers. Immigrants have a higher risk of work-related injuries than do natives. The available evidence supports the inference that immigrant workers are disadvantaged in terms of self-perceived health and mental distress compared with the native population. However, the evidence to conclude that the working conditions are a potential mediator of the association between immigrant status and these health outcomes is very limited. Nonetheless, a few studies from the Scandinavian countries support the idea that controlling for occupational factors may partly mitigate the differences in risk of sick leave and disability pension between non-Western immigrants and natives.

Knowledge of the working conditions and occupational health of immigrant and ethnic minorities is important for initiating preventive and integrational efforts. However, this is challenging because of shortcomings in the available data, heterogeneity of immigrant populations, uncertainty about the validity of instruments and the lack of prospectively designed cohort studies. These challenges underscore the importance of collecting information on working conditions and health more systematically, particularly among groups that are presumed to be at greater risk of being employed in high-risk jobs.

To understand further the associations between working conditions, health and immigrant status, and to facilitate cross-country comparisons in the European context, large-scale studies that focus on different aspects such as immigrants’ cultural and socio-economic backgrounds, language skills and time lived in the host country are needed, as are investigations that are culturally appropriate and use instruments translated into the mother tongue of the target groups of immigrants. Tools and procedures that include immigrants and ethnic minorities in the existing data collection processes, such as censuses, national statistics and health surveys are also needed.

Many aspects of working conditions and occupational health related to immigrant movements remain to be investigated. There are indications of the over-representation of immigrants in low-skilled, high-risk manual jobs, which require confirmation through the analysis of valid empirical data. In addition, there is a lack of information regarding unsettled and undocumented immigrant workers. This matter is complicated by short-term, circular and return migration, which creates difficulties for data collection and reliable assessment of occupational health issues among immigrant workers.

## Additional files

Additional file 1: Search profiles. (DOCX 15 kb)

Additional file 2: Working conditions and occupational health among immigrant workers: The data (authors; country, year of publication; aims of the study; study design; sample description, working conditions; health outcomes, summary of main results and general methodological comments) extracted from the articles. (DOCX 61 kb)
