# Supplementary material for: A systematic review of working conditions and occupational health among immigrants in Europe and Canada
Source: BMC Public Health. 2018 Jun 20;18:770. doi: 10.1186/s12889-018-5703-3 (PMC6011510; doi:10.1186/s12889-018-5703-3)
Supplement: Supplementary file 1 — Search profiles. (DOCX 15 kb) [file 12889_2018_5703_MOESM1_ESM.docx]

**Additional file 1: search profiles**

Medline/ Embase search profile:

Ovid MEDLINE(R) Epub Ahead of Print, In-Process & Other Non-Indexed Citations, Ovid MEDLINE(R) Daily and Ovid MEDLINE(R) 2000 to 2016 (searched January 2017).

String 1: immigrant population

exp "Emigration and Immigration"/ or exp Ethnic Groups/ or exp Minority Groups/ or exp "Transients and Migrants"/ or exp Undocumented Immigrants/ or Illegal migrant.tw. or Labour migrant.tw. or Migrant worker.mp. or Nomad*.tw. or exp "Emigrants and Immigrants"/ or foreign worker.tw. or foreign labour.tw. or migrant labour.tw. or irregular migrants.tw. or Seasonal workers.tw. or refugees.tw. or asylum seekers.tw. or migrant.tw. or foreign born.tw. or foreign-born.tw.

AND

String 2: Occupational exposure or occupational Health

exp occupational exposure/ or occupational exposure*.tw. or occupational risk.tw. or occupational hazard.tw. or exp industry/ or occupational group*.tw. or work-related.tw. or working environment.tw. or at work.tw. or work environment.tw. or exp occupations/ or exp work/ or workplace*.tw. or workload.mp. or occupation*.tw. or worke* or work place*.tw. or work site*.tw. or job*.tw. or occupational groups.tw. or employment OR worksite*.tw. or industry.tw. or exp occupational health/ or exp occupational injury/ or exp occupational diseases/ or Asthma, Occupational/ or accidents, occupational/ or occupational injuries/ or exp occupational medicine or employee health.mp . or industrial hygiene.mp. or industrial health.mp. or occupational safety.mp. or occupational disease.mp. or worker health.mp. or occupational health.mp

AND

String 3: country

exp scandinavia/ or exp "scandinavian and nordic countries" or exp denmark/ or exp finland/ or exp iceland/ or exp norway/ or exp sweden/ or exp scandinavia or exp denmark or exp finland or exp iceland or exp norway or exp Sweden or scandinavia.mp. or denmark.mp. or finland.mp. or iceland.mp. or norway.mp. or sweden.mp. or exp europe/ or exp austria/ or exp belgium/ or exp france/ or exp germany/ or exp great britain/ or exp greece/ or exp ireland/ or exp italy/ or exp luxembourg/ or exp netherlands/ or exp portugal/ or exp spain/ or exp switzerland/ or exp malta/ or exp united kingdom/ or exp estonia/ or exp latvia/ or exp lithuania/ or exp bulgaria/ or exp croatia/ or exp czech republic/ or exp hungary/ or exp poland/ or exp slovakia/ or exp slovenia/ or exp Cyprus/ or exp canada/ or europe.mp. or austria.mp. or belgium.mp. or france.mp. or germany.mp. or great britain.mp. or greece.mp. or ireland.mp. or italy.mp. or liechtenstein.mp. or luxembourg.mp. or netherlands.mp. or portugal.mp. or "scandinavian and nordic countries".mp. or spain.mp. or switzerland.mp. or malta.mp. or united kingdom.mp. or estonia.mp. or latvia.mp. or lithuania.mp. or bulgaria.mp. or croatia.mp. or czech republic.mp. or hungary.mp. or poland.mp. or slovakia.mp. or slovenia.mp. or Cyprus.mp. or canada.mp.

AND

Limit to abstracts and languages (English or danish or finnish or norwegian or Swedish)

**Search strategy adapted to Social Sciences Citation Index**

TOPIC: (asylum seeker* OR refugee* OR seasonal worker* OR irregular migrant* OR (foreign (worker or labour or born)) OR immigrant* OR emigrant* OR nomad OR migran* worker* OR labour migrant* OR (illegal near/2 migrant*) OR (undocumented near/2 immigrant*) OR migrant* OR minorit* group* OR ethnic group* OR (emigration* or immigration))

AND

TOPIC: ((industrial(hygiene or health)) Or (employe* health) OR (occupational(health or injury or disease or asthma or accident* or medicine or safety)) OR job OR (work(place or site)) OR workload OR (work or workplace) OR occupation* OR work* environment OR at work OR (work near/2 related) OR occupational group OR industry OR (occupational(exposure or risk or hazard)))

Indexes=SSCI Timespan=2000-2016

AND

CU=(Scandinavia or denmark or finland or iceland or norway or sweden or europe or austria or belgium or france or germany or great britain or greece or ireland or italy or luxembourg or netherlands or portugal or spain or switzerland or malta or united kingdom or estonia or latvia or lithuania or bulgaria or croatia or czech republic or hungary or poland or slovakia or slovenia or cyprus or canada or liechtenstein or luxembourg or netherlands or portugal or spain or switzerland)

And

Refined by web of science categories: ( public environmental occupational health or sociology or demography or psychiatry or anthropology or psychology multidisciplinary or psychology applied )
