# Supplementary material for: A systematic review of working conditions and occupational health among immigrants in Europe and Canada
Source: BMC Public Health. 2018 Jun 20;18:770. doi: 10.1186/s12889-018-5703-3 (PMC6011510; doi:10.1186/s12889-018-5703-3)
Supplement: Supplementary file 2 — Working conditions and occupational health among immigrant workers: The data (authors; country, year of publication; aims of the study; study design; sample description, working conditions; health outcomes, summary of main results and general methodological comments) extracted from the articles. (DOCX 61 kb) [file 12889_2018_5703_MOESM2_ESM.docx]

Additional file 2: Working conditions and occupational health among immigrant workers

| **Authors (reference)**  **Country**  **Year** | **Study aim** | **Design / data** | **Sample description** | **Working conditions** | **Health outcome** | **Summary of main results:** | **General methodological comments** |
| --- | --- | --- | --- | --- | --- | --- | --- |
| Aalto A-M et al. (10)  Finland  2014 | Comparing all immigrant physicians to a sample of native physicians regarding employment position, perception of work-related stressors and work-related well-being. | Cross-sectional survey, random sample (Finns), total population (migrants). Comparative study. | Immigrant physicians with completed medical education outside Finland, n=1292 Response, migrants: 42%, n=549 Response, Finns: 56%, n=3646) Data collection 2010. | Work related resources: Job control, team climate and organizational justice. Work related well-being: Risk of severe burnout, work ability. | N.A | No significant differences pertaining to job demands job control or social climate. Immigrant physicians reported more often a high risk of burnout (OR 1.46; 95% CI 1.16−1.85). | Includes the total cohort of immigrant physicians at a given time. Weaknesses are that the data are self-reported, and the response is quite low. |
| Agudelo-Suarez et al. (11)  Spain, 2010 | To assess the extent of presenteeism in a sample of Spanish-born and foreign-born workers. | A cross-sectional convenience sample study, | Immigrant workers in 4 cities, n=2434, response 55.8%. Comparison group of native Spanish workers, n=509, response 55%.  Data collection 2008-09 | Information on socio-demographic characteristics, migration process, employment and working conditions, physical and mental health. | Sickness presenteeism | Sickness presenteeism more prevalent among immigrant workers, especially those with less than two years in Spain (42% in Spanish-born and 56.3% in Foreign-born; (OR 1.77; 95% CI 1.24−2.53) | Non-random sampling and included a selected group of Immigrants. Non-validated measure of sickness presenteeism. |
| Agudelo-Suarez et al. (12)  Spain, 2013 | To assess changes in mental health in a sample of immigrant workers between 2008 and 2011, i.e. before and during the financial crisis. | Face-to-face (2008) and phone (2011) interview of 318 immigrant workers, 50% women and 84% under 45 years of age | Immigrant workers (Morocco, Ecuador, Romania and Colombia), in 4 cities, with (n=318) | Explanatory variables were a)Sociodemographic (sex, country of birth age, resident status) | Poor mental health (GHQ-12) | Change in prevalence of PMH was higher in men (OR 4.63; 95% CI 2.11−10.16). Subgroups of men showing the largest detrimental mental health effects were: unemployed, with low salaries (<1,200 euros) | Small sample size and lack of comparison group (natives). Some of those worst hit by the crisis may have returned to their home country or emigrated elsewhere. |
| Agudelo-Suarez et al. (13)  Spain  2011 | To analyze the relationship between immigrants workers´ perceived discrimination and self-reported health indicators. | A cross-sectional survey. Structured interview with previously tested instruments. | I Immigrant workers in 4 cities, Immigrant workers n=2434, response 55.8%. Comparison group of native Spanish workers, n=509, response 55%. Data collection 2008-09 | Perceived discrimination was determined by answers to the question: “Have you ever felt discriminated against? | Self-reported health Mental health (12-item General Health Questionnaire) | 73.3% of men and 69.3% of women reported discrimination due to immigrant status. Workplace-related discrimination was associated with poor mental health (OR 2.97; 95% CI 2.45−3.60), and self-reported health (OR 2.20 ; 95% CI 1.73−2.80). | Non-random sampling and the use of a wide definition of discrimination based on the interviewees’, own perceptions of discrimination. Spanish language requirement within the inclusion criteria. |
| Ahonen & Benavides (14)  Spain  2006 | Describe the number of occupational injuries and fatalities among foreign-born workers in Spain, compared to Spanish born workers | Social Issues registry of nonfatal and fatal occupational injury in insured workers. | Nationality from registry (Spanish workers vs foreign workers) Registry as of December 2003. | N.A | Occupational non-fatal and fatal incidents. | Non-fatal accidents Immigrant men (RR 3.9; 95% CI 3.9−3.9 Immigrant women (RR 5.4 ; 95% CI 5.4−5.5  Fatal accidents Immigrant men (RR 4.4 ; 95% CI 3.9−5.1 Immigrant women: (RR 6.0 ; 95% CI 3.6−9.6) | The data only covers insured workers, which leaves a large and probably more vulnerable group of immigrant workers aside. The data also lacks socio-demographic information, except gender and age. |
| Akhavan et al. (15)  Sweden  2007 | To examine work-related health factors of female immigrants in comparison to native Swedish females. | Cross-sectional, comparative study, using questionnaire. | Swedish and immigrant women working in a municipality (Immigrant women, n=617 and Swedish women n=1812), Response 57%. Data collection 2003. | The questionnaire consisted of 69 questions on employees’ psychosocial and physical working conditions. | N.A | Immigrant’s reported higher levels of ethnic discrimination (OR 2.0) and gender discrimination (OR 1.4), but not sexual harassment (OR 0.8) compared to natives, and more often reported temporary employment (20% vs 8%among native women). | A cross sectional study presenting crude comparisons of native and immigrant women. The items are not validated and poorly described. |
| Alexe DM et al. Greece (16)  2003 | Assess the profile of farm injuries in Greece. | Cross-sectional Register data (a database run by four major hospitals) | Total injured persons, in a five-year Data collection in 4 regions, n=4326. Native Greeks n=3896 Immigrants n=430.  All ages. | N.A | Main outcome was work injury, treated at a health care institution. | Occupational farm injuries tend to be more severe among migrant workers as hospitalization was necessary in 23% of them as contrasted to 14% among Greek farm workers. | The register is reported to be reliable. In the absence of a suitable control group odds ratios and actual rates could not be estimated |
| Bengtsson & Scott (81)  Sweden  2006 | Assess differences between the sickness benefit consumption of immigrants and Swedes | Register-based representative panel. Longitudinal and comparative. | A randomly selected sample of 110,000 native Swedes and immigrants from 16 countries during the Data collection 1968–1996. | N.A | “Excessive” number of sick days (estimates based on the amount of sickness benefits paid out in a given year). | Most immigrant groups had an Increased risk of sick leave compared to Swedes (range 2-7 times higher sick leave). Individuals who earned less than others with the same educational level did showed an excess risk of sick leave. | Analyses adjusted for socio-demographic variables and year since immigration Due to data limitations, this study does not differentiate between full and part-time employment. No data on occupation or working conditions |
| Bergbom S, Kinnunen U (17)  Finland  2014 | Investigate how co-worker relations are associated with psychological well-being and job satisfaction. | Cross-sectional survey conducted at one workplace (a bus company). | The participants were immigrant (N = 183) and native (N = 186) employees in a transport company in Finland. Response 46%. | Co-worker relations were measured with a composite score | Job satisfaction and psychological well-being (RAND 36-Item Health Survey) | Co-worker relations were positively associated with job satisfaction and psychological well-being among natives and immigrants. Among immigrants, co-worker relations with natives was related to job satisfaction and psychological well-being. | A cross sectional study with a rather low response percentage. The respondents worked in the same workplace and the majority of them in the same job. Data collection of data collection not reported. |
| Bergbom and, Vartia-Vaannen (18)  Finland  2015 | Primary aim: To examine if immigrants are more bullied than native at a workplace. | Cross-sectional survey conducted at one workplace (a bus company). | The participants were immigrant (n = 183) and native (n = 186) employees in a transport company in Finland. Response 46%. | Self-labelling method to measure exposure to bullying with a single item and a definition | N.A | 14.5% of the total cohort reported bullying at work. Among immigrants the prevalence of bullying was on average three times more than among natives (21.8% vs 7.6%, OR 3.4 ; 95% CI 1.8–6.6). Immigrants reported exclusion by not being listened to or ignored. | A cross sectional study with a rather low response percentage. The respondents worked in the same workplace and the majority of them in the same job. Data collection of data collection not reported. |
| Bhui, et al. (19)  U.K.  2005 | To assess perceived discrimination and its association with common mental disorders among workers in different ethnic group | Cross sectional survey, face-to-face interviews. | White British, n=514, workers in different ethnic group (n = 1540)  The primary data was collected in 1998-99 | Insults, generally, and at work, previous job denial, and unfair treatment at work | Common mental disorders (CMD) measured by Revised Clinical Interview Schedule. No of cases = 292 | Ethnic minority groups reported sig. higher levels of racial/ethnic discrimination. No sig. difference in prevalence of CMD. The risk of mental disorders was highest among ethnic minority individuals reporting unfair treatment (OR 2.0; ; 95% CI 1.2−3.2) and racial insults (OR 2.3 ; 95% CI 1.4−3.6) | Representative sample. The survey was cross-sectional, and reverse causality may be implicated. |
| Biering, et al. (20)  Denmark  2016 | The aim of the study was to compare the incidence of work injuries among different groups of immigrant workers with the native working population. | Population-based comparative register study on general working population. . | Registered work injuries in the district of Odense, n= 430000 persons. Danish natives, n=65020, EU15 (except Denmark), n=865, new EU, 12 countries, n=460. Other, n=1594.  Data collection:2003-2007 and 2008-2013 | Occupation (NACE) Injuries were related to low or high risk sector, age, sex and Data collection of injury. | The study measured work related injuries in high-risk and low-risk sectors. N = 68069 in 64001 persons. | Immigrant workers from the new EU countries and from the rest of the world had higher risk of work injuries compared with natives (OR 1.13; 95% CI 1.02−1.24, and (OR 1.56; 95% CI 1.48−1.64 respectively). Migrants over 30 years of age, migrants in low-risk sectors, and migrants who had been in Denmark < 3 years were at higher risk. | The categorization of broad industrial groups may preclude identification of differences in risk between migrants and natives. Only registered migrants are included, excluding illegal migrants, short-term migrants and commuters. |
| Borrell et al. (21)  Spain   2006 | To describe self-perceived health status among Barcelona residents born in Catalonia, in the rest of Spain and in foreign countries; and (2) to examine the role of social class and its mediating pathways | Cross-sectional comparative study. General pop. Study from 2000 Barcelona Health Interview Survey. | All working population aged 16-64 years were included in the study. N =4214 were included in the study. Native Catalonian, n=3106, born in other areas in Spain (i.e. internal migration), n=946 and foreigners, n=162. Data collection 2000-2001. | Work exposure measured was: type of contract (temporary or no contract/other), job insecurity (high/low), physical hazard (yes/no), psychosocial hazard (yes/no).) Household labour (hours/week, i.e double burden). | Self-perceived health (SRH) | Foreign women reported more temporary contracts (40% vs 27% natives), psychosocial hazards (41% vs 53% natives) and physical hazards (33% vs 38% natives). No sig. differences for men. Immigrant men, but not women reported poorer health compared to natives (OR 2.16; 95% CI 1.14– 4.10). Social class or working/living conditions did not explain this difference. | Small number of foreign immigrants and no information was provided about this group (e.g. years living in Spain, language skills). No information on non-response was reported. |
| Brekke & Schøne (82)  Norway  2014 | To examine whether health is a contributing factor to the immigrant-native sickness absence gap | A cross-section questionnaire survey on health— linked to register data sickness absence (N=14,114). | Oslo residents born in 1924, 1925, 1940, 1941, 1955, 1960 and 1970. 18,770 individuals (46 %) participated. Around 10 % of the sample were immigrants. Data collection 2000–2001 | Ten dummy variables for occupation industry; this is done using 12 dummy variables, based on two-digit NACE codes | Sickness absence of 17 days or more 2000 and 2001. Self-reported health (SRH) | Non-Western immigrants had higher incidence of long-term sickness absence than did natives. Men: On average 6.3 more sickness absence days. Controlling for occupation reduces the sickness gap by approx. 40 %. Women: 8.3 more sickness absence days. Controlling for occupation reduced the sickness gap to approx. 32%. | Lower response among immigrants. Register information on the total population of Natives indicates that the results for the immigrant-native sickness absence gap are quite comparable. Analyses adjusted for demographics and employment |
| Brekke et.al. (22)  Norway  2013 | To estimate differences in sickness absence during pregnancy among immigrant and native women. | Population-based cohort study.  Register data. Comparative study. | A total of 573 women employed prior to their pregnancies were included, 51% were immigrants. Data collection:  2008-2010. | Five dummy variables for Occupation categorized according to ISCO-08 Part-time/full-time work (10-50%, 51-80%, 81-100%). | Doctor-certified sickness absence (> 2 weeks full sickness absence) during first and second trimester in pregnancy. | Immigrant women had more weeks of sickness absence compared to native women (OR 1.2; 95% CI 0.35–2.08). Occupation was not related to sickness absence. | Broad occupational categories of employed limits the possibility to draw conclusions on the importance of occupational factors. |
| Carneiro et al. (23)  Denmark 2010 | To investigate differences in the associations between health indicators and sickness absence among elderly care workers | Cross-sectional comparative study. Data from the Danish Health Care Workers Cohort-Class of 2004 | Danish (n=2,831, response 66 %) and immigrant (n=290, response 54%) elderly-care workers. Data collection:2005 | N.A. | Sickness-absence General Health, Back Pain, Sleeping Problems | Immigrants reported worse health status, but significantly lower sickness absence than natives. Immigrants with poor self-reported health and sleeping problems had significantly lower risk of high sickness absence compared to Danes. | A large study consisting of all health care assistants and health care helpers graduated in 2004. A weakness was The heterogeneity of the immigrant sample and its small sample size |
| Carneiro et al. (24)  Denmark  2013 | To investigate the association between health and sickness absence among cleaners in Denmark | Cross-sectional study. Convenience sample of 9 companies. Comparative study. | Danish (n=144) and non-western immigrant (n=132) cleaners. In all 78% of the invited cleaners answered screening-survey, of those 47% were included in study. Data collection:2007-2008 | N.A. | Sickness-absence (registers) Self-reported health, body pain (TBP) and chronic disease, blood pressure (objective measure). | No significant differences in sick leave between non-Western immigrant and Danish cleaners. Immigrant women reported significant higher levels of overweight and body pain. No significant differences in SRH or chronic diseases. Higher blood pressure among Danes. | A homogenous study population (cleaners) minimize confounding. Health data from different source. Limitations are the cross-sectional design, the small sample size and the heterogeneity of the immigrant population. |
| Cayuela et al. (25)  Spain  2015 | Examine differences in self-perceived health and mental health between immigrant and native workers, and assess the contribution to health differences due to educational level, occupational social class, and occupational condition. | Cross-sectional survey, the Spanish National Health Survey. Comparative study | Immigrants born in low-income countries residing in Spain for at least 8 years (n=711) and native workers (n=7880) Response 71 %. Data collection: July 2011-June 2012 | Educational level: (university, secondary school, basic/no-schooling. Occupational social class: manual, non-manual. Occupational conditions: Work-related stress, job satisfaction, physical demands and employment conditions. | Self-perceived health (SRH) and mental health (General Health Questionnaire) | Immigrant women reported poorer mental health (OR 2.02; 95% CI 1.39–2.93) and self-perceived health (OR 2.64; 95% CI 1.77–3.93) compared to natives. No sig. differences observed for men. Occupational social class counted for 25% of the difference in poor SRH and 17.6% for mental health. The impact of occupational conditions was 3% for physical demands and 9.% for job strain | The study does not provide any information on how physical demands or job strain was measured, and cannot be used to assess exposure levels. Further limitations are the cross-sectional design and self-reported data. The only confounder taken into account was age. Response percentage not specified specifically for the study population. |
| Chen et al. (26)  Canada  2010 | The study investigates the prevalence of over-qualification in the three dimensions (education, experience and expectation). | Longitudinal survey of Immigrants to Canada (LSIC; Statistics Canada, 2007b). | Healthy with previous work experience before entering Canada. Working population. N =2685. Data collection:2001-2004 | Three dimensions of over-qualification, education, experience and expectation in relation to occupation. | Self-reported Health (SRH) Mental health. | Four years after arriving in Canada, 51.6% of immigrants were overqualified for their jobs based on their education levels. Respondents experiencing any dimension of over-qualification were significantly more likely to report a decline in mental, but not general health. | The longitudinal design of the study allowed assessing changes in immigrants’ health during the process of resettlement among skilled immigrants in Canada. No reference group based on natives. |
| Chowhand et al. (27)  Canada  2014 | The study examines the job satisfaction of immigrant employees compared to Canadian born employees | Workplace and Employee Survey. Representative sample of immigrant employees. | Non-western Immigrants (n =7580) and Canadian born (n = 49,344). Response 86% Data collection: 1999 – 2004. | Six occupation group variables, Irregular employment,  work experience | Job satisfaction | Average job satisfaction score was 3.2 for all employees and 3.1 for immigrants. Recent immigrants reported the lowest job satisfaction. | The study provides little data on working conditions. No confounding variables are considered. Immigrants arrived in 1966, in 1967-1989, 1990-2004). |
| Claussen et al. (28)  Norway  2009 | To investigate if higher rates of disability pensioning observed among immigrants from developing and Eastern European countries can be explained by differences in occupations, income levels, self-reported health and mental distress. | Survey data linked to data on disability pensions from the National registers. General working pop in the capital (Oslo) Comparative study. | General working population (n=11,072, response 49%) Immigrants (n =1130): western (47%), Eastern European (38%) and Developing countries (43%). Data collection: 2001- 2004. | Occupation. Work conditions (job control, shift work, income, and job strain). Health (mental distress and degrees of freedom). | Disability pension during. | Non-western immigrants, after controlling of age and gender had an increased risk of disability pension (OR 2.3; 95% CI: 1.6−3.2). Adjusting for occupation, job conditions and income, there was no longer a difference: OR 0.88; 95% CI 0.46–1.67. | Rather low response. The medical examinations may have attracted participants with poorer general and mental health among immigrants from developing countries. The collection of data takes place among inhabitant in the Oslo district; workers < 40 years are not included. |
| Connel et.al. (29)  Ireland  2007 | To investigate the nature of ocular injuries related to occupation in construction among non-nationals. It examines the use of ocular protection gear and attitudes towards eye safety practices. | Investigation of patients with eye injuries at an accident and emergency clinic in Dublin, Ireland. | Of 155 patients, 80 were Irish and 75 non-national, Data collection: 2003 - 2005 (two months at the Hospital clinic). | Enquiry about occupational injury, full ophthalmic medical examination and visual outcome. | Injuries involving penetration of eye injuries (4.9%) involved only non-Nationals. . | Among patients of construction-related eye injuries 48% were foreign-born. Their proportion among construction workers was reported to be 9%. Attendance at safety courses was 70% for non-Nationals and 90% for nationals. | A rather small sample and no information provided about the population at risk. No discussion of possible limitations. . |
| Cross and Turner (30)  Ireland  2012 | Examine the relationship between job satisfaction of relatively recent immigrants focusing on expectations and perceptions of fairness at Irish workplaces. | Cross sectional survey. Snowball sampling. Face-to-face interviews. | Immigrants: European Union countries (n = 480) Non-EU countries (n = 104). Data collection:2006 -2008 | Occupational status (measured as skills needed) Employed in home country (full time; part time; unemployed.) Measure of Fairness, and Met expectations | Satisfaction of work and overall sense of well-being. | Non-EU immigrants and those moving from high skilled jobs to low skill in Ireland was significantly more likely to report both distributive and unfairness at work. Measures of fairness and met expectations were significantly associated with satisfaction of work experience and well-being. | The use of the snowball sampling method could bias results as it prevents the randomization of groups of subjects. No comparative data on natives provided |
| Dahl et al. (83)  Norway  2010 | To study long term sick leave among immigrants and Norwegian natives. | Database, linking the National Insurance Administration, Statistics Norway and the Directorate of Labour. | N = 200 022 person aged 16-67 residing in Norway (a 10 per cent random sample), observed for 8.2 years. Country of origin classified into eight categories/regions.  Data collection:1992 - 2003 | A crude measure of occupational sector/branch | Register-based sick leave Data collection > 14 days or more | Immigrants from Asia (OR 1.5, p<0.01), Africa (OR 1.5, p<0.01, and South America (OR 1.1, p<.05) had more sickness absence than did native. Immigrants from Western Europe, North America, and Oceania had less sickness absence. Second-generation immigrants had the same level of sickness absence, as did natives. | State employees, farmers and persons who are self-employed excluded. Thorough adjustment for confounders, but no data on occupation or working conditions. |
| Davidson and Orr Ireland (31)  2009 | Investigate whether foreign-nationals were overrepresented in the Plastic Surgery service at the St James hospital in Dublin. | Case study of plastic surgery patients. | Foreign nationals from 27 different countries (n =81) Data collection: August 2006 to February 2007 | Industry sector: Construction, Education level and time in current employment | Work-related injuries (n = 201) | Work-related injuries: 40% of all injuries were among foreign nationals. Foreign workers had shorter time in current employment and higher percentage of serious injuries | No statistical analyses included – describe cases and observations without comparison with background population |
| DelAmo et al. (32) Spain  2010 | Describe the prevalence and the risk factors for poor mental health among female and male Ecuadorian migrants living in Spain compared to Spanish females and males. | Population based home survey (33 regions in Spain to represent different density levels of immigrants). . | Ecuadorian nationals (n = 568) and Spanish nationals (n = 1188 (Age: 18 – 55). (Response 69%) Data collection: January 2006 to January 2007. | Occupational factors: Employment Type of contract Work-dissatisfaction Atmosphere at work Individual salaries Education | Possible psychiatric case (PPC) GHQ-28 | Higher risk of PPC of Ecuadorian females than Spanish females (OR 1.6; 95% CI: 1.1–2.4). No sig. difference for men. Dissatisfaction of work, fourfold increase risk of PPC for both Ecuadorian and Spanish females. Having university studies doubled the odds of PPC in Ecuadorians. | Latin-American female interviewed Ecuadorian males. Thus cultural and gender differences could have had some effects on the results. Only registered migrants were included. |
| Diaz-Serrano (33) Spain  2013 | The study tests the preconditions for the competition between immigrant and native workers and investigates whether the quality of their jobs differ. | Health Survey of Catalonia 2006 (ESCA2006) Cross sectional design. Representative sample. Comparative study of native and immigrant workers in Spain. | Natives ( n = 6580 (Internal migrants (n = 1,850) Immigrants are foreign born workers n = 910 (Latin America, n = 380, EU-15 or other wealthy countries, n = 150, Africa, n = 260, Other countries, n = 120) | Weekly hours, working times, type of contract, working conditions: noise, dust, heavy loads, repetitive, monotonous, autonomy, alone, colleagues, superiors, discrimination, job satisfaction. | Job-satisfaction perceived as different tastes related to working conditions  Work injuries | Perceived discrimination: Africans: 28%, Latin Americans: 14.4 %, and EU15:4.9%. Africans employed in jobs with high injury risk 0.23 % vs. 0.12 % cent for EU-15. Compared to natives, the probability of having a permanent contract decreases about 33.1 and 37.8 per cent for Latin American and African workers, respectively. Africans also reported increased exposure to physical exposure (i.e. noise, dust, and heavy loads), poor relationships at work and monotonous work. Latin Americans reported increased risk of monotonous work. No sign. difference for job satisfaction. | Latin American and African immigrants, report an average length of stay of approximately 6.6 and 9 years, respectively. Results concerning irregular status of immigrants were considered impossible to retrieve by author due to missing data. |
| Dunlavy et al. (34)  Sweden  2016 | The study explores the association between the health status and educational mismatch among employed populations in Sweden. | Two general population surveys. Cross sectional Representative sample Comparative study | Native-born (n = 2359) / Western Europe (n = 589) / Outside Western Europe (n = 1200)  Response 61.5%, immigrant response 50%.  Data collection: 2010. | Objective (average education level by occupation – individual education levels). Subjective perception of necessary years of education) Employment status: Full/part-time work. | Self-reported Health (SRH) | Poor self-reported health: Native-born (18%) / Non-western (30%) Over-educated (objectively): 21 % non-western vs. 14. % among native-born workers. Objectively over-educated (OR 2.04; 95% CI 1.29–3.25) foreign-born workers from countries outside of Western Europe, greater odds ratios for poor-self rated health compared to native-born matched workers. | Foreign-born respondents in this study reported high levels of Swedish language proficiency, and the mean length of residence in Sweden was 22.3 years. No information on immigrants who had lived in Sweden for less than five years. |
| Dunlavy and Rostila (35)  Sweden  2013 | Study psychosocial and physical working conditions among native health outcomes among foreign background workers compared to that of native workers. | Two representative Surveys Cross sectional | Native Swedish (n = 2055),  Immigrants (n = 1966)  Living in Sweden more than 5 years Data collection 2010-2011 | Demand Control Model constructs with four proxy measures of psychosocial demands and decision latitude in the workplace. Physical working conditions: 1) ergonomic stress 2) physical demands | Health outcomes: self-reported health (SRH) and mental distress. | Immigrants reported lower decision latitude (59.7% vs 45% natives). No differences in job demands and social support. Immigrants reported higher levels of physically demanding work, but not ergonomic demands.  Significant increased risk of SRH and mental health compared to native Swedish workers: Eastern European (OR 2.39 and OR 2.05), Latin Americans (OR 1.5 and OR 1.85) and other non-western workers (0R = 1.79 and OR 1.50). Adverse working conditions only minimally influenced the risk of poor health | The foreign born workers included was a selected group staying in Sweden for a long time”. Excluded: self-employed and farmers. The study did not provide a statistical test for differences in working conditions between natives and immigrants, but provided an overall group test. Analyses already adjusted for occupational class before working conditions was entered into the model. |
| Dzurova and Drbohlav (36)  Czech Republic 2014 | Analyses the relationship between immigrants’ self-reported/rated health (SRH) and their perceived working conditions | Questionnaire surveys. A mixed sampling using different questionnaire surveys made in different years. | N=1498 working individuals (age 18–62 years) 38.1% from Ukraine: 61.9% natives.  Data collection:2008, 2012,2013 | A combination of four perceived work related factors (i.e. discrimination, violence/threats, job demands and risk of accidents= are defined as discrimination. | Self-reported health (SRH). | Poor SRH was reported by 23.6% of the sample, whilst females from Ukraine declared the poorest health (28.5%). Reported differences in workplace discrimination between Ukrainians and Czechs (males: Ukrainians 29% versus Czechs 4%; females: Ukrainians 38% versus Czechs 7%). | Survey on Czech-born from 2008. There is no statistical test of differences between immigrants and natives. A crude overall measure of work related exposure are presented in the analyses, which makes it difficult to interpret. |
| Elders et al. (37)  Netherlands  2004 | Study differences in disability rates among Turkish and Dutch Scaffolder | Register data Case-control Large company Comparative study | Turkish workers (born in Turkey or one Turkish parent (N=131 disability cases) Dutch (n = 125 disability cases) Response = n.a.  Data collection:1981 to 2000 | Years worked as a scaffolder | Registered with a disability pension scheme | Increases risk of disability scheme among Turkish scaffolder (RR 2.48 ; 95% CI1.94-3.18) | Data from a single company. Scaffolding is a small industry employing about 3000 persons. In the Data collection 1980 -85 45% were of Turkish origin, in the Data collection 1985-200 the proportion was only 16. |
| Font et al. (38)  Spain  2012 | To examine the relationship between immigration and mental health, taking into account psychosocial factors at the workplace | Cross sectional study Representative sample General working population Comparative study | Working population 16-65 years. Immigrant workers (n = 404) and n = 3699 native workers). Response = 60% Data collection:2004 to 2005 | The questionnaire included the 21 scales medium length version of the COPSOQ ISTAS21 psychosocial questionnaire | Mental health stratified into good and poor according to the median (SF-36) | Immigrants reported worse mental health than natives did (PR 1.09; 95% CI 1.02-1.16). Immigrants exposed to psychosocial factors reported worse mental health, but estimates were similar to those of exposed natives. | Comparison of mental health score not adjusted for age or gender. The survey was conducted during a Data collection of economic growth, before the economic crisis. Information about length of residence not available. |
| Font et al. (39)  Spain  2011 | To study differences between Spaniards and immigrants in exposure to psychosocial factors at work | Cross sectional study Representative sample General working population Comparative study | Working population 16-65 years. Immigrant workers (n = 404) and n = 3699 native workers). Response = 60%. Data collected 2004 to 2005 | Psychosocial factors ( quantitative demands, job control, possibilities for development, social support from colleagues, social support from superiors, insecurity) | N.A. | Immigrant were more often manual workers (89% vs 81% among natives). Immigrant manual works reported significant higher levels of exposure than did Spanish manual workers for 5 out 7 factors (OR ranging between OR 1.3 to OR 1.1). No sign differences reported for non-manual workers. | Representative sample of salaried workers in Spain. Immigrants were defined as ‘anyone living in Spain that was from any other country’, without differentiating between those from developing countries and those from developed countries. |
| Frickman et al. (40)  Switzerland  2012 | Study construction work accidents in a 10-year analyses | Register-based study based on data from an emergency department at a university hospital (covers 2 mill inhabitants). | Patients who reported their occupation as “construction worker” and/or identified the place of accident as a construction site (n = 782)  Data collected: 2001 -2011 | NA | Registered work injuries | The proportion of foreigners injured was 66.4% (i.e. more than twice the proportion of foreigners in the general working population). | The study had no information on the number of employees in construction sector and could not calculate incidence rates for Swiss and non-Swiss national. |
| Gamperiene et al. (84)  Norway  2007 | examined the association between psychosocial and organizational work  conditions and mental health among women employed in the cleaning profession. | Survey  Cross-sectional  representative of the cleaning sector | The participation  rate was 64% (; n = 374 Female cleaners; 86.3% of the women were born in Norway) | 26 questions was used to assess the psychosocial  work environment (QPS NORDIC) | Mental health (HSCL-25) | Immigrants reported significantly more mental health problems than did natives (OR 2.8; mean HSCL-25 scores were 1.62 and 1.37, respectively). Adjusting for working conditions did not reduce the difference in mental health. | Cross-sectional study. Small group of immigrant women.  Data collection of data collection not reported. No comparative data on working conditions presented. |
| Gil-Gonzalez et al. (41)  Spain  2014 | To study the prevalence of perceived racism/discrimination and its association with health outcomes | Cross sectional survey Representative sample Comparative study | Spanish born (n = 26054). Immigrants (n = 3344). 96 % of the total theoretical sample was interviewed. Data collected: 2006-07 | Perceiving racism at the work place. (‘during the last year experienced discrimination because of  his/her sex, ethnicity or country of origin, educational level or social class, sexual orientation, religion’’. | Self-reported health Mental health (GHQ-12) | Men: Spanish 0,1% vs. Foreign 5.7%, (OR 48.1 ; 95% CI 28.2-82.2)  Women: Spanish 0,1% vs. Foreign 5.0% (OR 43.5 ; 95% CI 25.5-74.3) | Ethnic minorities with Spanish nationality could not be differentiated from the rest of the native population. Association between work-place discrimination and health was not presented for immigrants separately. |
| Gravseth et al. (85)  Norway  2003 | To describe the epidemiology of occupational injuries in Oslo. | Patients´ records from an Accident and Emergency department in Oslo.  Patients with serious injuries were interviewed about the accident. | Occupational injuries treated by the emergency ward or ambulance Service were recorded over a Data collection of three months. Data collected: 2001 | Occupation | Registered occupational injuries | 1153 injury incidents were registered, 229 (20 %) of which were considered serious. Of those with serious injuries, 30 % had a non-Scandinavian language as their first language. Their proportion of the work-force was reported to be 12% | Occupation was unknown or not codified in 279 cases (24%). The occupational distribution among foreigners was not known. |
| Hansen et al. (42)  Norway  2014 | To examine whether occupational factors are associated with non-western immigrants’ greater sickness absence | Register-data Representative, census based comparative | N = 2.2 mill individuals, observed for 3.3 y. Country of origin: Norway, Nordic countries, Western Europe. Eastern Europe, North America and Oceania, Asia including Turkey, Africa, South and Central America. Data collected: 2003-2006 | Occupation (4 digit ISCO 88) , exact matching | Sick leave 16 days or more | Immigrants from outside Western Europe had more sickness absence than did native Norwegians. Adjusting for occupation reduced the observed difference in risk of sickness absence by 12 % (Eastern Europe) to 26% (Africa) and reduced the difference in average number of sick leave days by 7% (south America) to 29% (Africa). | No data on occupational factors but occupation. There may be differences within occupation on 4-digit level. Thorough adjustment for confounders (age, gender, education, fulltime work, income, refugee sending country) No data on sick leave shorter than 16 days |
| Hogh et al. (43)  Denmark  2011 | To explore whether immigrant health workers are more at risk of bullying than Danish staff members | Survey data Representative of health care students Prospective study comparative | N = 5,635. Of these, 89% were Danish and 10.4% were immigrants. (2.4% “western” and 8.1% and ‘‘Non-Western’’ countries)  Data collected: 2004 | Bullying: one question about ‘‘bullying’’ behavior at work and the self-labeling type was chosen. To | N.A | Bullying at work within the past 12 months, 8.5% among the Danes, 12.9% among the Western and 15.2% among the Non-Western respondents. Controlled for gender, age, type of job, and type of education showed that the Non-Western immigrants had an overall 85% higher risk of reporting bullying. | Adjusted for age gender previous exposure to bullying and type of job. Lower response in general among immigrants at T2 – 67% of the Danish respondents compared to 54% of the immigrants. |
| Hoppe (44)  Germany  2011 | To investigate differences in psychosocial stressors and resources between immigrant and German low-wage workers, and (2) to examine group differences in their association with well-being | Cross-sectional Convenience sample comparative | Employees from a mail service company n =89 immigrant workers and n = 146 natives. | Seven scales measuring psychosocial working conditions were selected from previously validated job analysis instruments | Psychological distress. Psychosomatic complaints | Immigrants reported higher level of exposure for two out of seven stressors (i.e. more conflicts with co-workers and supervisors and verbal aggression from customer) than natives did. No significant differences for job distress and psychosomatic complaints. Stressors more strongly associated with distress among German workers | Immigrant workers had lived in Germany, on average, for 15 years. Immigrants and natives were in comparable job. Small sample and low external validity. Relevant estimates not adjusted for confounders. Data collection Data collection not reported. |
| Johansson et al. (45)  Sweden  2012 | To study whether the impact of employment/unemployment on health differs among (documented) immigrants in comparison to natives. | Register-based longitudinal and comparative cohort study. Used registers: three annually updated databases. | Immigrants legally immigrated since 1960, who in 1990 were 28-47 years, registered: as residents in Sweden (n = 243 869). Data collection 1990-2008. | Employed/self-employed vs. unemployed by register-based data in 1990 | Disability pension, mortality Hospitalization for lung, heart, psychiatric, and musculoskeletal disorders. | All immigrants had a higher risk for disability pension than natives did. Men: (HR 1.9; 95% CI 1.9–2.0) / women HR 1.7; 95% CI 1.7–1.7). Non-Nordic immigrants had mostly equal or more favorable outcomes as regards diagnoses and mortality than natives. | Representative sample and adjustment of relevant confounders (age, white/blue collar, educational level, income + time of residence). Limitations: exposure to work conditions not known. |
| Jönson and Giertz (46)  Sweden  2013 | To investigate whether immigrant (born outside Sweden) care workers perceive their situation at work to be more problematic than their native peers do. | Cross-sectional survey. Randomly selected members from registers from unions. Comparative study. | Swedish-born care workers (n = 626, response 66.6%) Immigrant) care workers (Nordic countries (n =31) and non-Nordic countries (n = 56). Data collection in 2005. | Thirteen variables measuring psychosocial factors (e.g. work load, insecure employment) and 5 concerned problematic relations incl. discrimination | N.A | Immigrants from non-Nordic countries perceived themselves more often exposed to having a high workload (3.3 times). Non-Nordic men experienced five times more prejudice and women two times more than natives did. | The validity and reliability of measures used are not described. The separate responses of immigrants and natives not known. The sample consisted of union members. |
| Jorgensen et al. (47)  Denmark  2011 | To investigate differences in objective and self-reported health measures between immigrant and Danish cleaners. | A cross-sectional comparative study of immigrants and natives (working as cleaners). | 179 immigrant (= born in non-Western countries.) and 166 native cleaners from 9 workplaces. Immigrants. Data collection 2007-2009. | N.A | The Work Ability Index, Self-reported health (SF-36), musculoskeletal problems. Objective: BMI (>= 30), blood pressure. | Immigrants reported poorer self-reported health (SRH = 46% vs. 38%, p < 0.01), more musculoskeletal pain and reduced work ability (57% vs. 42%, p < 0.01) than the Danish cleaners did. No differences in chronic diseases, or regular use of painkillers. Natives were more often current smokers, had high blood pressure and drank more alcohol than did immigrants. | A small study of immigrants and natives working with the same kind of job (cleaners). Unclear whether health differences can be attributed to working conditions and/ or other differences- The findings pertain mostly to women, as the number of men was small. |
| Krings et al. (48)  Switzerland  2014 | To investigate whether immigrants report more experiences of workplace incivility than do natives, to explore the relation between selective incivility and perceived discrimination. | Cross-sectional survey study. A randomly selected sample | 1359 natives and 302 immigrants, employed and between 25-55 y. Immigrants were mainly from Germany or France and south/ north Europe. Data collection 2012. | Perceived discrimination based on ethnicity or nationality during the past 12 months(single item) | N.A | Being a German/French immigrant (OR 13, p<.001) or an immigrant of another nationality (OR 7.3, p<.001) increased the probability of reporting having been discriminated against. | Systematic selection of immigrants (needed sufficient proficiency in French or German to participate). |
| Kuusio et al. (49)  Finland  2013 | Study intention to leave the job among I foreign-born and native GPs , and it associations to (stressors at work) at work with | Cross sectional comparative survey study. Target samples: Random sample of natives and all immigrants. | Native physicians (n = 3787, response 56%). Immigrant physicians (n = 553): 44% from Russia 26% from Estonia 30% others, response 43%.  Data collection: 2010. | Psychosocial factors: job demands (5 items stress index). Patient-related stress. Stress related to teamwork (4 items). High job control | Outcome: Intention to leave (single item, to switch to another physician’s job). | Immigrants intended to leave their job (59%) more often than natives (52%) did. Associations between psychosocial factors and intention to leave were more consistent among native doctors. | A representative cross-sectional with Adjustment for relevant confounders. The study does not really shed light on whether the working conditions for immigrants and natives differ. |
| Manstrangelet.al.  (50)  2010  Italy | Estimate the rate of work-related accidents among employees without a legal employment position and/or regular working visa in an area of North Eastern Italy | Patients´ records from an Accident and Emergency department combined with population data on foreign-born residents in the hospital area, and the estimated population of illegally workers. | 419 patients´ records were collected. Of these, 146 raised suspicion: reported to have happened at home, but the type of injury did not correspond the actual description. Data collection 2004. | The number of the illegally employed non-EU workers were the denominator of the rate | The 146 injury cases were the numerator of the incidence rate | Injury rates for illegally employed immigrants varied from 109.1 to 271.8 per 1,000 non-EU illegal employees, compared to 65 per 1,000 reported in the general working population in 2004. | According to the authors, there is an incentive for over-reporting accidents at home rather than at the workplace in the Italian insurance system. Judgment on workplace versus home-related injuries was assessed based on secondary information about the industry and the nature of the accident. |
| Miller & Travers (51)  UK  2005 | Examine the mental well-being and job satisfaction of minority ethnic teachers in the UK | nationwide cross-national questionnaire study (with both open and closed questions) | N = 208 minority ethnic teachers (Response 9%), of which 160 (77.7%) were women. (55.8%, n=115) were born overseas, mainly from countries in Asia and Africa. Age range: 21-65 | Sources of stress: 31 items Ethnic discrimination: 14 items Coping strategies: measure by Daniels, Harris and Briner, 2001 | Mental ill-health: (GHQ-12) Job Satisfaction Scale | Overseas teachers experienced more ethnic discrimination than their UK counterparts did. Ethnic teachers reported poorer mental health and lower job satisfaction compared with other groups (another teacher study, general population). | A cross-sectional study with very low response and small sample size. “Sources of stress” measure self-developed. Data collection not reported. |
| Niewenhuijsen et al. (52)  Netherlands  2015 | Examine whether mental health inequalities between ethnic groups are mediated by exposure to unfavorable working conditions | A cross-sectional questionnaire study Randomly sampled Part of a large-scale multi-ethnic cohort study | Ethnic groups living in Amsterdam: 1355 Dutch (n = 1355) Ethnic minority groups (n = 4923). Overall response about 27.2%.  Data collection started in 2011, includes questionnaire data collected until December 2013 | Working conditions: work-related recovery opportunities scale (i.e. opportunities for time off the job and possibilities to control rest breaks and interruptions during work day Perceived stress at work (1 item) | Mental health (MCS) Depressive symptoms(PHQ-9) | All ethnic minority groups reported higher prevalence of lack of recovery opportunities: 27% to 42% compared to 17% in Dutch workers. The distribution of perceived work stress showed a mixed pattern. Most ethnic minorities reported sign. poorer mental health. The higher risk of mental health problems in ethnic minority groups was partly accounted for by a lack of recovery opportunities at work, but not by perceived work stress. | A cross sectional study with a low response. Mental health was defines as a linear outcome variable. Considerable sample size mainly well-validated, reliable measures. No measure of working conditions. |
| Olesen et al. (53)  Denmark  2012 | To investigate differences between non-Western and Danish cleaners pertaining to psychosocial work environment. | Cross-sectional study Comparative | 148 Danes and 137 non-Western Immigrants From nine workplaces in Denmark.  Data collected: 2007-8 | Scales from the short version of the Copenhagen Psychosocial Questionnaire (COPSOQ) using ordinal logistic regression. | N.A | Non-Western immigrants reported a sig. better psychosocial work environment on a number of scales (i.e. higher scores with regard to recognition (OR 1.92), quality of Leadership (OR 1.81), trust regarding management (OR 1.72), and justice (OR 2.14) | A cross sectional study with a small sample size. Respondents from the same low-skill trade were compared, which minimized confounding. |
| Olesen et al. (54)  Denmark  2012 B | Compare the association between psychosocial work environment and hypertension among non-Western immigrant cleaners and Danish cleaners. | Cross-sectional study Comparative data | 148 Danes and 137 non-Western Immigrants From nine workplaces in Denmark.  Data collected: 2007-8 | Psychosocial work environment measured with scales using The Copenhagen Psychosocial Questionnaire (COPSOQ). | Hypertension (based on blood pressure measurement and/or currently using antihyper­tensive medicine. | Non-Western immigrant: no sig. relationship between poor psychosocial work environment and hypertension. Danish cleaners: high trust regarding management and high predictability were associated with low prevalence of hypertension. | A cross-sectional study and rather small sample. Both objective measures and self-reported data on health. The COPSOQ questionnaire may have limitations when used among immigrants. . |
| Ortega et al (55)  Denmark  2010 | Examine the psychosocial work environment, and the health and well-being of Danes and immigrants working in the Danish elderly care sector. | Cross-sectional survey Comparative data | Elderly care workers in 36 Municipalities (n = 9949; Danes=7101 Western immigrants=184, Non-western immigrants=124. Response 79% for Danes and 69% for immigrants. Data collected 2005. | Questions about the position and its activities; work hours; psychosocial work environment, Physical work environment; violence, threats, bullying and sexual harassment | Depression symptoms, quality of sleep, client-related burnout), well-being, Sickness absence; physical capacity. | Non-western immigrants perceived the work environment more positively than Danish colleagues, but scored lower on possibilities for development. Non-western immigrants reported significantly more depression symptoms, poorer quality of sleep and more burnout than Danish colleagues did. Associations between psychosocial work characteristics and health and wellbeing were stronger among Danes than among immigrant. | A large-scale survey of the elderly sector in Denmark.  immigrant workers in the elderly care were younger; tended to be employed more often as health-care helpers and had been employed at their current work place for fewer years than Danes. |
| Pasca & Wagner (56)  Canada  2011 | To explore occupational stress, mental health and satisfaction (life, job, relationship) as experienced by immigrant individuals attempting to achieve integration into Canadian workplaces. | Cross-sectional Respondents selected from a contact list and by snowball sampling Self-report questionnaire | 42 employed Canadian born (CB) and 42 employed Non-Canadian born (NCB) working in education, health care, and social services. Response =85%.  Data collected: | Demographics Measure of occupational stress. | Mental health. Measure of job satisfaction | There was no difference between NCB and CB regarding occupational stress and overall satisfaction with job. non-Canadian born workers in the fields of education, healthcare and/or social work report more similarities than differences when compared to the responses of Canadian born workers | Cross sectional survey with a small sample size. NCB professionals who immigrated into Canada under the economic category. Data collection Data collection not reported. |
| Perez-Carceles et al. (57)  Spain  2014 | To iden­tify workers with a hazardous drinking problem by means of a self-reported questionnaire | Cross-sectional study | A random sample of 385 immigrant workers from North Africa, South America, India-Pakistan and other countries. Data collected: 2010-2012. | Demographic variables (sex, industrial branch, country of origin, years of residency, living conditions | Hazardous drinking AUDIT (≥8) and/or CDT (>2.6) | 13.8% (n=53) were screened as hazardous drinkers and 53.8% (n=207) were teetotalers. Risk factors were: working in the construction industry or agriculture), being resident in Spain for more than 7 years | Cross sectional study and no reference data was provided for natives. The sample comprised mainly men. |
| Pikhart et al. (58)  Czech republic 2010 | To explore job satisfaction and self-reported health among immigrants with illegal/irregular status and immigrants with legal status | Two cross-sectional questionnaire surveys | 285 Post-Soviet and Vietnamese immigrants (126 legal and 159 illegal/ irregular). Data collected 2003 to 2006. | Satisfaction with work | Self-reported Health (SRH) | Non-significant difference in SRH among illegal immigrants compared to legal immigrants in the fully adjusted analysis (OR 1.50; 95% CI 0.92–2.45). Not satisfied with work played a role for the poorer SRH for all groups. | Relatively small sample and not representative sample. No work exposures and no information about occupation. No reference data on natives. |
| Premji et al.  (59)  Canada  2010 | To assess the potential linkage between the proportion of immigrants in jobs and the level of risk associated with these jobs. | Register study aggregated at the level of job categories. | Register data on accepted claims in combination with census data (20% and national survey data. Data collected:2000 | Job categories (manual, mixed, non-manual) | Injury and illness frequency rates | Immigrants and members of ethnic and linguistic minority groups in Montréal are over-represented in jobs with a high level of risk for being compensated for work-related injuries or illnesses. More so for women than for men. | Risk defined on the basis of accepted claims, aggregated data only. Crude occupational categories may hide differences between natives and immigrants. |
| Premji & Lewchuk (60)  Canada  2014 | To examine disparities in hazardous employment characteristics and working conditions among Chinese and white workers in Toronto, Canada | A population-based survey  Representative Comparative | 831 Chinese and 780 natives were included in the preliminary analysis.  Data collected: 2005-2006. | Heavy workload, temperature, air quality, noise, toxic substances. Discrimination, harassment, stressful work, | N.A | Discriminatory treatment more prevalent among Chinese (37% vs. 19% natives). There were no statistically significant differences between Chinese and whites in type of employment variables (i.e. temporary contract, working hours Chinese workers sign. more likely to report heavy physical workload. Among men, natives were more likely than Chinese to report exposure to toxic substances. No difference for other physical working conditions. | Cross sectional study. Analyzes adjusted for length of time in Canada, age, and education and. work sector. Higher educational level among the immigrant group. |
| Premji & Smith (61)  Canada  2013 | To examine the association between education-to-job mismatch and work injury. | A cross-sectional study Representative Comparative | General working pop. > 25 years who had been employed at some point in the previous 12 months (N= 63462)  Data collected 2003 and 2005. | Current occupation, educational level, Education-to-occupation mismatch. Recent and non-recent immigrants compared. | Work-related injury, Repetitive movement injury (RMI) | Over-educated recent immigrants reported increased risk (RR 3.25; 95% CI 1.19 8.83) of work injuries requiring medical attention compared to non-recent immigrants not over-educated. No significant differences were observed for other immigrant groups. | Current occupation used to establish education to- occupation mismatch categories. Adjustment for working conditions was done using broad categories of physical demands. No comparative data on injury rates among natives. |
| Robert et al. (62)  Spain  2014 | To evaluate the influence of changes in employment conditions on the incidence of poor mental health in the context of an economic crisis. | Follow-up survey was conducted at two time points, | Convenience sample (n = 2434, n = 29%) of immigrant workers Sample = 216 subjects who reported good mental health in 2008 , were interviewed again in 2011  Data collected: 2008 and 2011 | Employment contract, monthly net income, legal status, social security registration, employment status, Working weekly hours | GHQ-12 (dichotomized into ‘good mental health’ and ‘poor mental Health) | Risk factors for poor mental health: loss of job (OR 3.62; 95% CI 1.64–7.96), increased working hours (OR 2.35, ; 95% CI: 1.02–5.44). Increased risk of poor mental health among people whose legal status was temporary or illegal and decreased risk among those being registered in the Social Security system in 2011. | Longitudinal study, but a small sample size and only those with sufficient skills in Spanish included. Attrition bias. Non-participants in the second survey may have been affected by adverse economic and employment situations. |
| Ronda et al. (63)  Spain  2013 | To compare self-reported exposure to occupational health risks in foreign-born and Spanish-born workers in Spain. | Cross-sectional study. Convenience sample  Comparative  . | N = 1,841 foreign-born (the four countries with the greatest number of Nationals) The sample of Spanish-born workers (N = 509, 51.5 % males) was constructed to resemble the foreign-born sample in terms of sex, age (20–40 years old) and area of residence.  Data collected: 2008 | Exposure to safety risks, chemicals, physical contaminants, physical load and psychosocial agents). Each risk was rated on a frequency scale (never, rarely, sometimes, often and always), assuming a normal working day. | N.A | No significant differences were observed for mechanical loads (i.e. carrying heavy load and postures) or for Chemicals/physical agents (noise, working with chemical or inhalation of dust/gases). Foreign-born men in non-services sectors and manual occupations reported lower exposure to occupational risks than did Spanish workers. Foreign-born female workers were more likely than Spanish workers to report working many hours/day and exposure to extreme temperatures. | Large comparative survey. Sampling took place in neighborhoods where at least 15 % of the residential population is foreign-born. Immigrant group had lived in Spain for at least one year; worked in Spain for at least three months; and having adequate Spanish language abilities to participate in the interview. |
| Ronda et al. (64)  Europe  2012 | To determine immigrant workers’ exposure to occupational risks and compare it with that of non-immigrant workers in Europe. | Cross- sectional European survey (EWCS 2005) Comparative | 1000 economically active inter­viewed in 2005 in each of 31 European countries.  In total, N = 501 immigrant men and 424 immigrant women.  Data collected: 2004-5 | Working hours, contract, chemicals (breathing fumes, dust or powders), physical agents (noise, vibrations, temperatures), physical load and psychosocial conditions (working at very high speed and shift work). | No outcome Migrants compared to Non-migrants (male and female, respectively) | Male immigrant manual workers report a greater number of negative physical and mechanical working conditions. Female immigrant manual workers are three times more likely to not having a contract and 2.5 times more likely to carry and move heavy loads. | Small number of immigrants in the dataset, not possible to analyze data according to region of origin. Little information about immigrants. No information about length of residency. Different response between countries. |
| Saeed et al. (65)  Ireland  2010 | To investigate whether recent socio-demographic changes and recent health and safety measures have impact on the trends of ocular trauma in the South East of Ireland. | Retrospectively patient material Register covering all patients admitted to a Department of Ophthalmology | N = 517 patients were admitted with ocular trauma.  Data collected October 2001 and September 2007. Focus on 2006/2007, following the influx of immigrant workers. | Patients’ demographic details, including nationality, mechanism of injury and nature of injury were recorded. EU Accession States (EUAS) | Acute ocular trauma | Significant higher incidence among immigrants (EUAS: 134 per vs 100.000 and 10 per 100.000 among Irish). EUAS workers employed in the construction and/or manufacturing industries had a fourfold risk of hospitalization because of ocular injury when compared to Irish workers in the same sectors. | Data were retrospectively assessed and may have been inaccurately recorded. Limitations inherent in the collection of census data, as people illegally resident in a country may fail to complete forms or may  give inaccurate info |
| Salminen et al. (66)  Finland  2009 | The aim of this study was to examine whether immigrant workers have a higher injury frequency compared to Finnish workers when performing the exact same tasks under the same working conditions. | Cross-sectional study. Comparative data. | A total of 176 Finnish and 130 immigrant bus drivers. Response rate 40%. Data collection: 2005-6. | Bus drivers in a transport firm | Self-reported occupational injuries during the past 12 months and n = 134 injuries reported to an insurance company. | The injury rate (injuries per 1,000 employees) was higher for Finnish drivers (113.6) than for immigrant drivers (77.5). The mean of absence days was 15.8 per injury for both groups. Based on the questionnaires, immigrant bus drivers were slightly but not significantly more often involved in occupational injuries than Finnish drivers (13.0% vs. 9.8%) did | A cross sectional study with a low response. Two different sources of data on injuries. Comparison between Finnish and immigrant injury reports with same work tasks limits confounding. |
| Salvatore et al. (67)  Italy  2013 | To compare the occurrence of work-related injuries among immigrants in Italy to that among Italians, to evaluate potential risk factors, focusing on the construction sector. | Cross-sectional general population survey. | N =60,528 individuals (response was 89.9 %.), of whom 2,195 (3.6 %) were immigrants from High Migration Pressure Countries (HMPC)  Data collection:2007 | Immigrants from High Migration Pressure Countries (HMPC) | Self-reported work related injuries | The age-adjusted OR of injury for immigrant men, compared to Italian men, was 1.82 (95 % CI 1.53–2.16), the odds was higher among immigrant construction workers (OR 2.05; 95% CI 1.56–2.69) and unskilled construction workers (OR 8.64; 95 % CI 2.85–26.20) compared to Italian workers in the same occupational group. No differences  between immigrants and Italians were found in other categories,  .No associations were found among women. | The survey  included legally residing immigrants who had registered as  residents in the town where they lived, who have achieved  a higher level of integration; the sample size of immigrants was not sufficiently large to  analyze industrial sectors other than construction |
| Sattler et al.  (68)  Ireland  2009 | The present study focused on this new, rapidly growing patient subpopulation presenting with hand injuries to the Department of Plastic Surgery in Cork University Hospital (Ireland) from 2000 to 2005. | All hand injuries presenting to the Dep. Of Plastic Surgery July-December 2005 were analyzed and compared with data for the previous 5 years. | Of special interest were patients from the 10 countries that joined the EU on the May 1, 2004.  Data collection:2005 compared to the previous five years | Nationality | Hand injuries to the Dep. Of Plastic Surgery (N = 762 patients) | The total number of patients with hand injuries remained relatively stable, ranging from 798 in 2000 to 762 in 2005. Comparing 2003 with 2004, the patient numbers of the EU group more than doubled from 18 (2.4%) to 41 (4.9%). Almost three-quarters were construction workers (52%) and factory workers (20%). | This is a purely descriptive study. No information about the population at risk, and little information about working conditions and occupation. |
| Shields and Price (86)  U.K  2002 | To investigate perceived racial harassment at the workplace and it impact on job satisfaction and quitting behavior | Cross sectional survey of British National Health Service Nurses | Eligible sample n = 1203 nurses of non-white ethnicity, aged 21-60. Black Caribbean (38%), Black Africans (27%), South Asian (16%), South East Asian (19%),  Data collection:1994 | Racial harassment from colleagues (single item) Racial harassment from patients (single item) | Job satisfaction Intention to quit | 40% and 64% of ethnic minority nurses reported racial harassment from colleagues and patients, respectively. Perceived discrimination was associated with low job satisfaction, which in turn, was associated with intentions to quit job. | Cross-sectional association study. Little information about immigrant status (i.e.  Self-reported ethnicity as other than white). Poor description of the measurements. |
| Sieberer et al. (69)  Germany  2011 | To determine the incidence of depressiveness in a large multi-ethnic working population with and without a history of migration. | The cross-sectional study asked employees of a university hospital to complete a self-rating question- naire. | N = 2932 person (41.7% response).  In all, N = 419 reported a history of migration (275 (first-generation (M1) and 143 second-generation (M2) migrants.  Data collection:2008 | Sociodemographic data, migration status and indicators of acculturation | Depressiveness (CES-D). | According to the CES-D scores, 8.7% of non-migrants (suffered from clinically relevant depressive symptoms, compared to 16% of the M1 group (OR 2.10; 95% CI: 1.44–3.04) and 14% in M2 (OR 1.68; 95% CI: 1.01–2.79). | Cross-sectional study Large study but very little information on work environment (model adjusted for occupation - that showed no effect on risk for outcome) |
| Smith et al. (70)  Canada  2009 | To examine the proportion of a recent cohort of immigrants to Canada who were working in jobs that were more physically demanding than those in which were they worked before migration to Canada. | Cohort of immigrants to Canada with occupational position measured before and 2 and 4 years after arrival in Canada. | A representative sample of immigrants, aged 15 and older, who arrived in Canada.  Data collection between Nov 2000 and Dec 2001. | Worked before immigrating and were working when re-interviewed 2 (N=4331) or 4 (N=4238) years after arrival in Canada. | Employment in an occupation with higher physical demands than that employed in before arrival in Canada. | Respondents most likely to be employed in more physically demanding occupations both 2 and 4 years after arrival in Canada were those with poorer English language skills, those with lower levels of education and those coming to Canada as refugees. | A large representative sample shows the bad language skills and refugees have higher physical demands at work. |
| Smith and Mustard (71)  Canada  2009 | To examine the burden of work-related injuries among immigrants to Canada compared to Canadian-born labor force participants. | Cross-sectional study  Self-reported Comparative | Data from the 2003 and 2005 Canadian Community Health Surveys. General working population sample (N=99 115). Sample of immigrants in their first 5 years in Canada (N=2859; 3 and those in Canada for 6–10 years (N=3013) | N.A | Self-reported work related injury – total and activity limiting | Immigrant men in their first 5 years in Canada reported higher risk of injuries that required medical attention (OR 2.08; 95% CI: (1.02 to 4.25) compared to Canadian-born respondents. No excess risk was reported among female immigrants compared to Canadian-born female labor market participants. | Large cross-sectional study with adjustment for relevant confounder.  Crude measure of immigrants, sell-reported belonging to a visible minority. |
| Smith PM and Mustard (72)  Canada  2010 | To examine the prevalence of occupational health and safety risk factors among immigrants to Canada compared to Canadian-born labor force participants. | Cross-sectional study, self-report comparative | Statistic Canada’s Survey of Labour and Income Dynamics (N =15,000 households, cross-sectional response in 2001 reported 79.1%). Immigrant category (21 y. + N= 1811, 11-20 y N = 699, 6-10y N = 294 and <= 5Y N = 298). | Working conditions: non-membership in a union; physically demanding occupations; workplace with less than 20 employ; regular shift work; irregular shift work; and having non-permanent employment. | N.A | Recent immigrants (< 10 years in Canada) were more likely to be working in physically demanding occupations and small workplaces. Immigrants in their first 5 years in Canada had a higher probability of working in temporary employment. Immigrants who had been in Canada for up to 20 years were still less likely to be members of a union or collective bargaining agreement. | Large study with adjustment for relevant confounder. Limited the possibilities to examine specific differences among different groups of immigrants. Crude measure of immigrants, self-reported belonging to a visible minority. |
| Solé et al. (73)  Spain  2013 | To assess disparities between immigrants and natives in the role played by working and contractual conditions, particularly risk exposure, in determining the occurrence of disability | Register: an administrative dataset, 4% random draw from a reference population, consisting of employed workers (wage earners and self-employed) and people on benefits. | 718,958 observations, 21-64 years old, who has contributed to the Social Security (SS) system ≥5 years (mandatory for workers in Spain), with an active record in MCVL in 2006: 681,078 natives, 37,880 immigrants. | Working conditions: - Temporary contract - Self-employed - Low-skilled job - Years since enrolment in SS | Disability Permanent disability of any kind between 1980 and 2006.  High-risk job = top quartile Injury and illness rates by industry–occupation (440 job–industry cells). | Immigrants more likely to be employed in high risk jobs (3.9%-points higher than natives), , but there are differences by birthplace Immigrants had a lower probability of receiving disability pension (1.6% vs. 4.9 natives) and higher probability of temporary contracts (48% vs. 37%) and low-skilled jobs (35% vs. 28%). In both groups, temporary employment and low-skilled jobs increased the risk of disability. | Only insured immigrants were included - could lead to conservative estimates. No information about baseline health. Bureaucratic processes to obtain disability pension - difficult for immigrants to apply, may lead to lower estimates for immigrants. |
| Soler-Gonzalez et al. (74)  Spain  2008 | Study incidence and duration of sick leave among immigrants and native-borns in Spain | Prospective, convenience sample (Patients treated in primary care) | 1427 immigrants and 2793 Spanish natives treated at 5 primary care centers in the city of Lleida. Data collection: 2005, followed for 6 months. | N.A | Sick leave: - Risk - Incidence - Duration - Diagnosis | All sick leave outcomes were lower among immigrants. 6-month risk: 12.7% vs. 19.5%. Incidence (per 100 person-years): 32.5 vs. 43.3 for men 18.6 vs. 35.6 for women. | No work-related data (type of work, contract, and work exposures). Included only patients, not general working population. Only workers enrolled in Spanish social security program, not undocumented workers. |
| Sousa et al. (75)  Spain  2010 | To analyze the relationship of legal status and employment conditions with health indicators in foreign-born and Spanish-born workers. | Cross-sectional. Quota sampling. Questionnaire-based interview. Sampling of Spanish-born in neighbourhoods with ≥15% foreign-born. | 1,849 foreign-born workers ≥1 year in Spain, worked ≥3 months, adequate Spanish language. 509 Spanish-born workers. All 20-40 years. Response: 55.8% and 55.0%. Data collection: 2008-2009. | Legal status: - Documented (=work permission) vs. Undocumented employment cond. (=contract): - Permanent vs. Temporary vs., no contract. | Self-reported health (SRH) Mental health (MH; GHQ-12) | Contract type significantly related to poor health in both foreign-born and Spanish-born workers. Associations were not significantly different for Spanish born and foreign- born workers with the same contract type. Compared with Spanish-born, with permanent contract the highest risk was reported for: undocumented foreign males, <3 years in Spain: SRH: OR 2.68 (1.09-6.56). MH: OR 2.26 (1.15-4.42) and foreign females, no contract, >3 years: SRH: OR 4.63 (1.95-10.97). | Large sample of undocumented immigrants, with combination of legal status and employment conditions. Cross-sectional limits the interpretation of the results and no data on occupation or working conditions. |
| Subedi and Rosenberg (76)  Canada  2014 | To examine whether there are differences in socio-economic characteristics and health outcomes of recent and more established immigrants in Canada. | 2 surveys, Canadian Community Health Surveys (CCHS) 2001 and 2010. | Immigrants with residency in Canada <10 years (N=3708) and with residency in Canada >10 years (N=6956).Data collection: 2000-2001 and 2010). | Residency in Canada <10 years vs. >10 years. Work-related stress: - Self-perceived work stress (5 cat) - No. of hours worked/week | Self-reported health: 5 categories, made dichotomous, good vs. poor health | Sign. difference in the SRH of immigrants with <10 years vs. >10 years of residency in Canada: Poor health: 7.2% vs. 17.5%. Work-related stress (not at all): 13.6% vs. 11.5%. ). | Most analyses were not adjusted, not for age or other confounders. No relevant comparison group |
| Sundin et al. (87)  Sweden  2011 | Study burnout among foreign-born (FBW) and native Swedish women (SNW) | Longitudinal panel survey | 3616 women at T1 (2002), 2300 women at T2 (2003), 427 FBW, 1873 SNW living in the county of Stockholm. | Job demands, job control, social support (18 items), and working hours. | Burnout: (SMBM): 2 subscales: emotional/physical exhaustion (8 items), cognitive weariness (6 items). | Foreign-born women reported higher level of burnout than Swedish native women, mean 3.2 vs. 3.0 at T1 did. Job demands and working hours were associated with burnout among Swedish native women. Similar estimates reported for immigrant women. | High non-response (T1: 35.9% in total pop, 79.1 among FBW. T2: 32.2% non-response). Based on self-reported data. Questionnaires only in Swedish. Did not consider country of origin or residence time in Sweden. |
| Sundquist et al. (77)  Sweden  2003 | Analyze associations between work factors (psychological job demands, decision latitude, and social support) and long-term illness among foreign-born and Swedish-born employed people. | Cross-sectional, combining 4 annual simple random samples from the Swedish Annual Level of Living Survey. | 10,072 Swedish-born, 710 labor migrants, 333 refugees, employed, aged 25-64 years.  Data collection:(1994-97 | Psychosocial working conditions: - Job demands - Job decision latitude - Social support (based on 2+7+2 items) | Self-reported long-term illness (LTI). | Refugees had higher risk (OR 1.33; 95% CI 1.05-1.69) of LTI than Swedes, no sig difference observed for labor immigrants. 63% of refugee’s manual workers had low decision latitude vs. ca. 45% in the other groups. Small differences in job demands between the groups. No interaction between migration status and job strain or low control. Refugees with low work-related social support had a high risk of long-term illness. | Cross-sectional, with self-reported data and possible cultural bias. Comprehensive study with low non-response, representative of the whole population. Adj. for age, sex, marital status, SES. Did not consider country of origin or residence time in Sweden. |
| Thurston and Verhoef (88)  Canada  2003 | To address the extent of injury among immigrants and the possible determinants of these injuries | Questionnaire survey  convenience sample identified through service agencies in a large urban center in Canada | The sample consisted of 532 workers who had been in Canada for a mean length of 6.45 years. Data collection: 1994. | Five industries:  processing (32.8%), service (26.2%), cleaning (19.4%), fabrication  (13.4%), and construction (7.0%). | Lost-time injury rates were calculated as (lost-time injuries/person years) x 100. | The lost-time injury rate for immigrant workers was 6.0. The lost-time injury rate for the province of Alberta was 3.6 %.  Length of stay, a proxy for acculturation, had no relationship to injury, but working in a language other than English did. | The sample was both self-selected and included selected industries known to have high risk. The population at risk was not known. The comparison with "reference" data may not be comparable. |
| Tiagi (78)  Canada  2015 | To examine the gap in injury and fatality rates between immigrant men and women and their Canadian-born counterparts | Linked survey and register, both data from 2011 | General population survey (2.7% of Canadian pop) Sample: 709,449 observations, age ≥15 years.  Data collection 2011. | Canadian-born vs. immigrants, grouped in 5 categories according to years in Canada). Data for both industry and occupation is listed at the 2-digit level, (i.e. 19 industries and 30 occupations) | Injury rates per 10,000, fatality rates per 100,000. The injury or fatality rate in individual i’s occupation or industry. | No sign. difference in occupational injury and fatality rates between various arrival cohorts of immigrants and Canadian-born workers, both women and men. Industry injury and fatality rates are lower for the most recent arrival cohorts vs. Canadian-born workers. | The register does not include all workplace injuries, only accepted time-loss injuries. Injuries among self-employed are often not reported. |
| Tiagi (79)  Canada  2016 | To analyze whether there are intergenerational differences in occupational injury and fatality rates among the first, second and third (or more) immigrant generations in Canada | National Household Survey (NHS) and publicly available 2011 from the Association of Workers’ Compensation Boards of Canada | 887 012 records (2.7% of the Canadian population). First generation (born outside Canada). Second generation = born in Canada with at least one parent born outside. Data collection: 2011 | Occupations | Aggregated data on : injury rate and fatality rate (per 100 000 persons), by gender, div | Second-generation immigrants worked in less hazardous jobs compared with the first generation (RR, injury rates = 1.06, 1.05–1.07 RR, fatality rates = 1.07, 1.05–1.10) and compared with the third (or more) generations (RR, injury rates = 1.08, 1.07–1.09 RR, fatality rates = 1.16,1.14–1.18) | No reference data on injury rates in the total population. The results are based on imputed injury data at occupational level and may be subject to misclassification. information about occupational coding is not provided |
| Tora et al. (80)  Spain  2015 | To explore the effects of 2008 economic crisis on the prevalence of adverse psychosocial working conditions among Spanish and foreign workers. | 2 cross-sectional surveys  Comparative study | 2007 survey:  9080 Spanish,  1322 foreign workers. 2011 survey:  7652 Spanish,  786 foreign workers. | Working conditions:  - Psychological job demands - Job control - Social support - Job insecurity | N.A | Low job control and low social support more prevalent among foreign workers than nationals.  Both Spanish and foreign workers perceived higher job insecurity in 2011 vs. 2007 (PR=2.47 and 2.44, respectively). | Survey in Spanish language. Non-response was not reported. No information on item covering psychosocial conditions. No formal statistical test for differences reported |
| Vives et al. (89)  Spain 2013 | To assesses the association between precarious employment and poor mental health using the multidimensional Employment Precariousness Scale. | Cross sectional survey Representative nationwide gen. pop. comparative | Wage earning population 16-65 y living in Spain (N = 7650) (Spaniards N = 6221 and immigrants N = 556) response was 60%. Conducted in 2004-5 | Employment precariousness scale (26 items). (employment instability, individual-level bargaining over employment conditions, low wages and economic deprivation, and limited workplace rights and social protection) | Poor mental health (SF-36 mental health scale. | Prevalence of poor mental health: Women: 21.9 % (Spaniards) and 33.1 % (Immigrant), p<0.01. Men: Prevalence 29 (Spaniards) and 32.7 (Immigrant) =, p=0.28 Associations between EP and mental health is reported but not separate for immigrants. | The prevalence estimate was stratified by gender but not adjusted for age or any confounders. The restriction to employees with a contract may limit the generalizability of the result |
| Vives et al. (90)  Spain 2011 | To determine the prevalence of precarious employment cross social groups including immigrants in Spain | Cross sectional survey Representative nationwide gen. pop. comparative | Wage earning population 16-65 y living in Spain (N = 7650) (Spaniards N = 6221 and immigrants N = 556) response was 60%. Data collection 2004-5 | Employment precariousness scale (employment instability, individual-level bargaining over employment conditions, low wages and economic deprivation, and limited workplace rights and social protection) | Poor mental health (SF-36 mental health scale) | Prevalence of employment precariousness: Spaniards 5.6% and immigrants 18.3%). Prevalence rate ratios of poor mental health was 1.9 and 1.4 for women and men. Population attributable risk of poor mental health to employment precariousness was higher among immigrants | Definition of precariousness is based on a rather arbitrary cut-off (>=2 on a scale range 1-4) Excluded self-employed and those without a contract. The association between precariousness and poor health was not estimated separately for Spaniards and immigrants. |
| Wadsworth et al. (91)  U.K.  2005 | To determine levels of work stress in three ethnic groups, and assess the association between work stress and well-being | Cross-sectional survey, through face-to-face interviews. | White British, N= 216, Black Caribbean’s, N=206, Bangladeshi, N=206. Inclusion: in work aged 18-65 years, self-reported ethnicity. Data collection: 1998-99 | Effort-reward Job strain Unfair treatment discrimination | Work stress (1 item) | Discrimination at work: 12% (n = 24) of black African–Caribbean, 7% (n = 14) of Bangladeshi and 6% (n = 14) of white respondents (P = 0.09). High work stress: White British, (8%), Black Caribbean’s 18% Bangladeshis (6 %) (P<0.01). | Representative sample. The survey was cross-sectional and reverse causality may be implicated. Not adjusted for possible confounders. |
